# Reproduction of melting behavior for vitrified hillforts based on amphibolite, granite, and basalt lithologies

**DOI:** 10.1038/s41598-020-80485-w

**Published:** 2021-01-14

**Authors:** John S. McCloy, José Marcial, Jack S. Clarke, Mostafa Ahmadzadeh, John A. Wolff, Edward P. Vicenzi, David L. Bollinger, Erik Ogenhall, Mia Englund, Carolyn I. Pearce, Rolf Sjöblom, Albert A. Kruger

**Affiliations:** 1 School of Mechanical and Materials Engineering, Washington State University, Pullman, WA USA; 2 Materials Science and Engineering Program, Washington State University, Pullman, WA USA; 3 Department of Materials Science and Engineering, University of Sheffield, Sheffield, UK; 4 Pacific Northwest National Laboratory, Richland, WA USA; 5 School of the Environment, Washington State University, Pullman, WA USA; 6 Museum Conservation Institute, Smithsonian Institution, Suitland, MD USA; 7 The Archaeologists, National Historical Museums (SHM), Uppsala, Sweden; 8 Luleå University of Technology, Luleå, Sweden; 9 US Department of Energy, Richland, WA USA

**Keywords:** Mineralogy, Solid Earth sciences, Materials science

## Abstract

European Bronze and Iron Age vitrified hillforts have been known since the 1700s, but archaeological interpretations regarding their function and use are still debated. We carried out a series of experiments to constrain conditions that led to the vitrification of the inner wall rocks in the hillfort at Broborg, Sweden. Potential source rocks were collected locally and heat treated in the laboratory, varying maximum temperature, cooling rate, and starting particle size. Crystalline and amorphous phases were quantified using X-ray diffraction both in situ, during heating and cooling, and ex situ, after heating and quenching. Textures, phases, and glass compositions obtained were compared with those for rock samples from the vitrified part of the wall, as well as with equilibrium crystallization calculations. ‘Dark glass’ and its associated minerals formed from amphibolite or dolerite rocks melted at 1000–1200 °C under reducing atmosphere then slow cooled. ‘Clear glass’ formed from non-equilibrium partial melting of feldspar in granitoid rocks. This study aids archaeological forensic investigation of vitrified hillforts and interpretation of source rock material by mapping mineralogical changes and glass production under various heating conditions.

## Introduction

### Vitrified hillforts

Hilltop fortifications are a hallmark of Late Bronze Age and Iron Age civilization in continental and island Europe, with many thousands being known^1^. About 200 of these are referred to as “vitrified^2^” including ~ 3% of those in Scotland^3^; that is, some of the rocks used in the fortifications have melted and upon cooling formed glass which often fused adjacent rocks. Considerable study has been given to vitrified hillforts, particularly in Scotland^4,5^, with fewer but still significant studies in France^6^ and Sweden^7^. Examples of vitrified hillforts are also known from England^8^, Germany^9^, Portugal^10,11^, and occasionally other locations (e.g., Ireland, Isle of Man, Orkney, Shetland, Wales, Bohemia/Czechia)^12^.

First reported in the 1700s^13^, debates about the cause of the vitrification at these sites began from the earliest reports^14,15^ and continue to this day. Interpretations include destructive burning by enemies^1,2,4,16^, incidental melting due to signal fires^17^, lightning strikes^7^, deliberate melting for constructive mechanical enhancement^18,19^, or ritual destruction (e.g., decommissioning of a site)^6^. Although it is now apparent that there are doubtless multiple correct explanations for the appearance of this ancient glassy material, varying considerably by site^7^, the most broadly accepted general explanations are: (1) destruction by enemy fire and (2) deliberate firing by locals for construction. It has been argued^7,20^ that each site should be considered independently, and wide generalizations are not prudent. For instance, the Swedish vitrified hillfort Broborg has been interpreted as being vitrified deliberately for constructive fortification^2,7,21^.

Laboratory experimental studies melting putative source rocks^8,20,22^, geochemical and mineralogical investigation of archaeological materials^19^, and large scale demonstrations^7,16,17,23–25^ have attempted to settle the debates about vitrified hillforts in general, but much remains obscure and enigmatic^20^. Based on these multiple lines of evidence, most researchers believe the necessary temperatures range from 1000 to 1250 °C^6,8,18,21^ and depend only weakly on lithology^26^. However, temperatures as low as 850–900 °C are effective for melting certain rocks if time at temperature is maintained long enough^26^, due to a biotite-quartz eutectic^27^; Other studies cite long times and higher temperatures, up to 10 h at 1050 °C^8^. One recent study showed evidence of mechanical strengthening due to vitrification, potentially supporting a constructive hypothesis^20^. Detailed knowledge of the temperatures and times required to produce the observed morphologies and mineralogies of the vitrified rock at a given site form part of the evidence needed to distinguish between the various hypotheses of motivation for the vitrification. For example, if required temperatures are very high (i.e., > 1200 °C) and times long (i.e., days), it might be assumed that an enemy would be less likely to expend such extreme effort to burn a fort to the extent required for such melting.

### Broborg

In the current study, we investigate these debates in light of new evidence from Broborg (‘bridge fort’ in Swedish), located about 20 km southeast of Uppsala, Sweden (17.9515° E, 59.7556° N), and documented as vitrified since at least 1744^7,28^. Several previous researchers have investigated the vitrified fort at Broborg, most notably that of Kresten et al.^2,7,21,29,30^, with some recent preliminary and confirmatory studies looking at details of the vitrified material itself, as part of an ongoing project investigating vitrified hillfort glass stability over time^31–33^.

**Figure 1:**
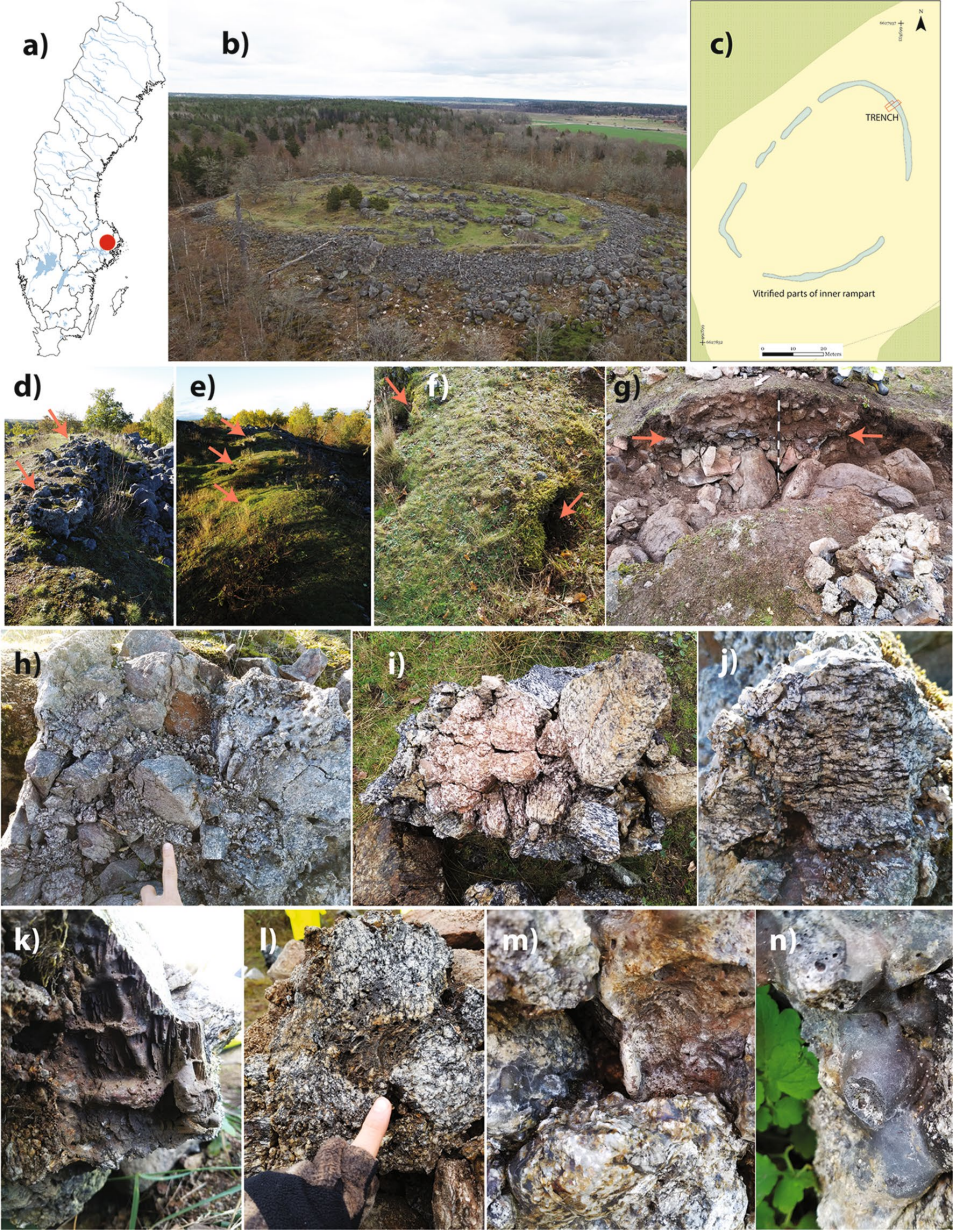
Broborg vitrified hillfort site: (**a**) location of the site within Sweden; (**b**) aerial view looking east; (**c**) plan map of the vitrified inner walls at the summit; d) ‘box-like’ construction on surface; (**e**) ‘box-like’ construction sub-surface; (**f**) ‘box-like’ wall section showing opposite side holes interpreted as used for firing; (**g**) 2017 excavation trench, with arrows showing vitrified portion; (**h**) multiple fused rocks; (**i**) rock with ‘golden biotite’ formed due to heating > 500 °C; (**j**) granitoid with ‘weeping’ dark melt from melted mafic minerals like biotite, indicating > 1000 °C; (**k**) charcoal or wood impressions in ‘dark glass,’ which are c. 2–4 cm on a side; (**l**) hottest part of melted granitoid from wall interior; (**m**) surficial ‘clear glass’; (**n**) amphibolite melt ‘drips’. Maps in (**a**) and (**c**) created using Adobe Illustrator 2019, modified after Englund et al.^34^; (**b**) from Englund et al.^34^, open access per CC BY 2.5 SE; all other photographs (**d**–**n**) by J. McCloy.

Figure 1 shows the main features of this site, including its location in Sweden (Fig. 1a), aerial view (Fig. 1b), aerial plan of vitrified regions (Fig. 1c), and recent excavation trench (Fig. 1g). Also shown are the main architectural features of the vitrified areas, known as box-like or cell-like features, approximately 2 m long × 1–1.5 m wide, interpreted as the units by which the wall was deliberately vitrified^7,34^. These boxes can be observed above ground-level around some of the site (Fig. 1d), while their location can be inferred due to surface topography in other parts (Fig. 1e). Finally, visible in most box sections are holes on opposite sides (Fig. 1f), interpreted as being locations for introducing a heat source and for placing a forced draught^34^—i.e., a bellows, as commonly practiced in contemporaneous bloomery iron smelting^35^.

Visual inspection (Fig. 1h–n) and previous work^21^ at the Broborg site suggest that both granitoid and amphibolite rocks are fused and have seen different degrees of heat. The result at Broborg is a heterogeneous artificial conglomerate of fused and melted rocks. Glasses of two distinct compositions have been reported, one more felsic, known as “clear glass” and one more mafic, known as “opaque” or “dark glass”^7,32^.

In the current work, we use a cross-disciplinary approach, and combine evidence from recent geochemical and mineralogical investigations of archaeological materials, field evidence from a 2017 excavation^34^, and laboratory melting studies of possible source rocks and related lithologies. The goal of the experimental study was to characterize the crystal phases in the archeological materials and compare them to possible source rocks melted in the laboratory under a variety of conditions. In this way, it was hoped that some constraints on temperature, oxygen fugacity, and cooling rate could be applied and compared to the observed solidus and liquidus behavior of the heated rocks and the mineral phases observed, as a function of the heat treatment conditions.

## Results

### Synopsis of experiments and analysis

For this study, vitrified rocks obtained from the Iron Age Broborg hillfort site were compared to laboratory-heated rocks of various types. X-ray diffraction (XRD), optical microscopy, and analytical electron microscopy—scanning electron microscopy (SEM) with backscattered electron (BSE) or energy dispersive spectroscopy (EDS) plus electron probe microanalysis-wavelength dispersive spectroscopy (EMPA-WDS)—were used to study the samples in this work.

Amphibolite, dolerite, and granitoid rocks were obtained from near the Broborg site. For comparison, basalt rocks were also studied, as they are commonly found at other hillfort sites. Details of the rock selection are provided in the “Methods” section.

Rock fragments were heated to different maximum temperatures then quenched and investigated, primarily using XRD. Variations were made by changing the starting rock size, cooling rate, and presence of carbon or clay. Select rocks were studied using ex situ XRD (i.e., heat treated then cooled to room temperature and measured) as well as in situ XRD (i.e., measured during heating and/or cooling). These results were compared with equilibrium mineralogical calculations using MELTS software^36–38^. Further details on the experiments and analysis procedures are provided in the “Methods” section and Supplementary information.

### Broborg hillfort vitrified rock samples

Two archaeological rock samples from the vitrified inner wall of Broborg were obtained as previously described^32^, from areas similar to those shown in Fig. 1g. Sub-samples of these rocks were denoted Bro-W, a largely white rock, and Bro-B, a mostly black rock (Fig. S1).

**Figure 2:**
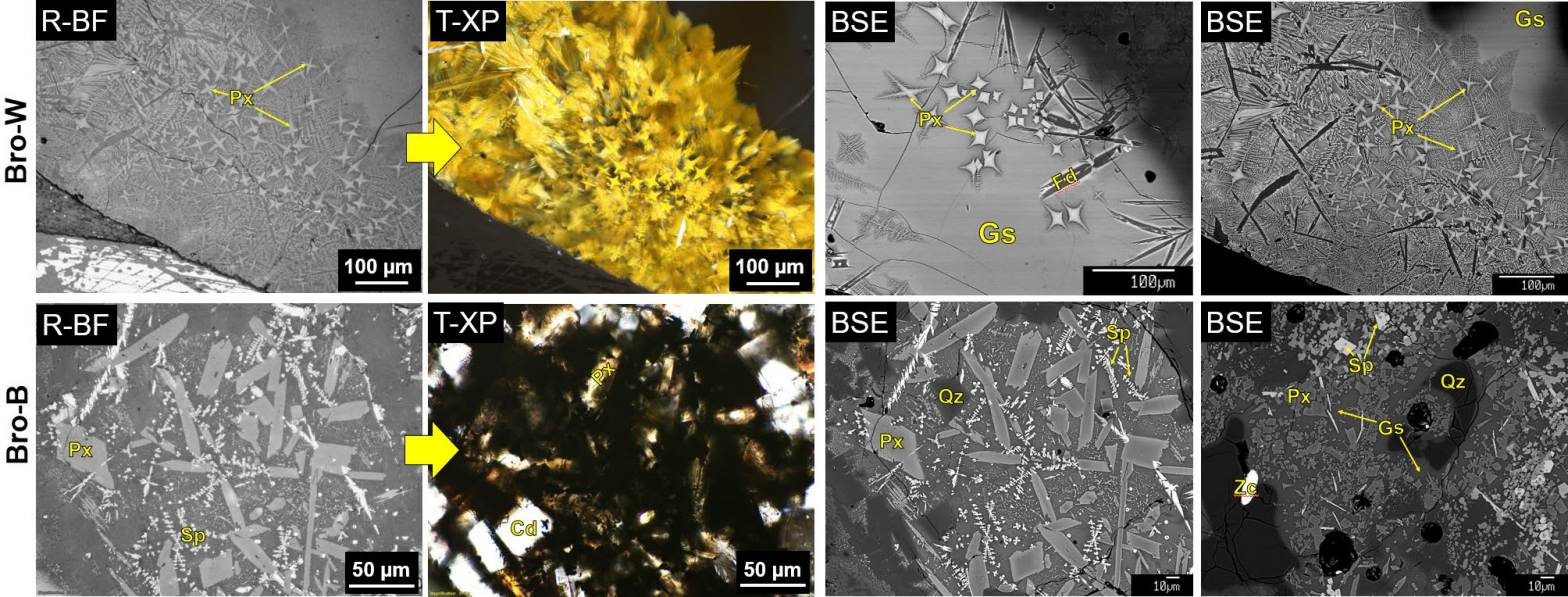
Typical microstructural features of archaeological samples Bro-W (top) and Bro-B (bottom). Reflected light bright-field (R-BF) and transmitted light cross polarized (T-XP) optical and scanning electron microscopy (BSE). Shows pyroxene (Px), spinel (Sp), cordierite (Cd), quartz (Qz), feldspar (Fd), and zircon (Zc) as well as glass (Gs).

**Figure 3:**
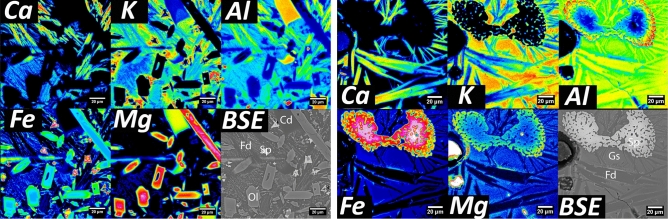
Microchemical imaging of vitrified hillfort samples. EPMA-WDS maps (left) Bro-B exhibiting cordierite (Cd), spinel (Sp), and olivine (Ol); (right) WDS Bro-W exhibiting feldspar (Fd) and relict spinel (Sp) which begun dissolving and exhibits compositional gradients. Red is high concentration and blue is low concentration.

For Bro-W, XRD identified feldspar, pyroxene, quartz, and spinel, although the specimens exhibited heterogeneity. Thin section optical microscopy (Fig. 2) showed feldspar needles, pyroxene, quartz, and spinel (both dissolving and reprecipitated from melt), with sizes and morphologies consistent with rapidly quenched conditions. Optical and electron microscopy (Fig. 2) and EPMA maps (Fig. 3) showed clinopyroxene dendrites (~ augite), feldspar needles (~ anorthoclase), dissolving spinel (hercynite-magnetite, with Mg–Al–Fe zoning), dissolving apatite, and olivine (~ forsterite, two morphologies, chunky and herringbone dendritic).

For Bro-B, powder XRD identified olivine, clinopyroxene, feldspar, magnetite, and cordierite, again depending on the sample, as confirmed by microscopy in Figs. 2, 3, and Figs. S2 and S3. In SEM, similar crystalline phases were found as in Bro-W, with the addition of accessory zircon and ilmenite. Cordierite is a low pressure, high temperature phase often seen in contact metamorphism or assemblages of pyrometamorphic materials^39^.

Minerals and textures observed in these samples match those described previously at Broborg^21^ and other vitrified hillforts with different starting lithologies^40^, with the exception of the identification of cordierite, seen here in Broborg samples for the first time, and confirmed in this study by both XRD and EPMA. Glasses around crystals have low SiO_2_ contents (49–66 wt%), and regions exist where high SiO_2_-felsic and low-SiO_2_ mafic glasses meet abruptly at an interface (Fig. S4).

### Source rocks

Whole rock chemistry (Table S3), thin sections (Figs. S7–S9), and XRD results (Section S3) on source rocks are provided in the Supplementary.

Amphibolite rocks from outcrops near Broborg (here denoted BA1-BA5) consist of (wt%) quartz (5–38%), plagioclase feldspar (22–39%), amphibole (~ actinolite, 5–67%), and biotite (~ phlogopite, 7–30%). Mineral contents of the rocks are broadly consistent with the definition for amphibolite rocks, defined by a region in the quartz-plagioclase-amphibole ternary, with a maximum of 25% quartz^41^, with the exceptions of BA1-BA2 which had high quartz and low amphibole. From thin section analysis, minor phases such as clinochlore, ilmenite, apatite, and zircon are also seen.

The dolerite, found as a dike running through the hill of the Broborg site, consists of (wt%) 70% amphibole, 26% feldspar, and 4% chlorite. Minor quartz and chlinochlore can be seen in thin section.

Granitoids are loose rocks of variable size, locally abundant on the outer, non-vitrified ramparts, and were presumably placed in ancient times. These rocks are glacial till, and highly variable. Mineralogically, they consist of (wt%) 25–28% quartz and 72–73% total feldspar—half or less as microcline K-spar with balance of plagioclase. Some show evidence of heating and recrystallization (Fig. S8).

A basalt is also included in these studies, since some well-known vitrified hillforts used basaltic source materials—notably Camp de Péran, France^6^, Dunagoil, Scotland^42^, and Bremerberg, Germany^12^. The basalt used here (Umatilla Member, Saddle Mountains Formation, Columbia River Basalt Group, Washington, USA^43^) had a starting phase fraction (wt%) 37% glass, 19% clinopyroxene (augite) and 44% feldspar (total albite/plagioclase + microcline). This initial amount of glass is consistent with the 10–60% range previously reported for Umatilla basalt^43^.

### Heat-treated rocks

#### Thermal analysis

Thermal analysis scans were obtained for amphibolite and dolerite rocks. All amphibolites exhibited similar features on heating (Fig. S10). Detailed studies of heating and subsequent cooling were conducted on BA5 amphibolite and dolerite, as high amphibole and low quartz contents suggested these might melt the easiest. Overall, the two rocks show similar thermal behavior (Fig. S11). Both samples exhibit ~ 3% weight loss with major water losses at ~ 550 °C and 1100 °C, due to breakdown of chlorite and amphibole (e.g., tremolite dehydrates 750–1000 °C), respectively^44,45^. The subtle endothermic feature seen in diagrams of both samples ~ 880 °C is likely related to melting of small amounts of chlorite and/or biotite. There are also two melting endotherms ~ 1100 °C and 1200 °C, assigned to melting of amphibole and plagioclase feldspar minerals, respectively^46,47^. Upon cooling, there is an exothermic peak at ~ 1100 °C for both samples, attributed to crystallization of pyroxene. These results are consistent with the in situ hot-stage XRD results below.

#### Phase transformation

**Figure 4:**
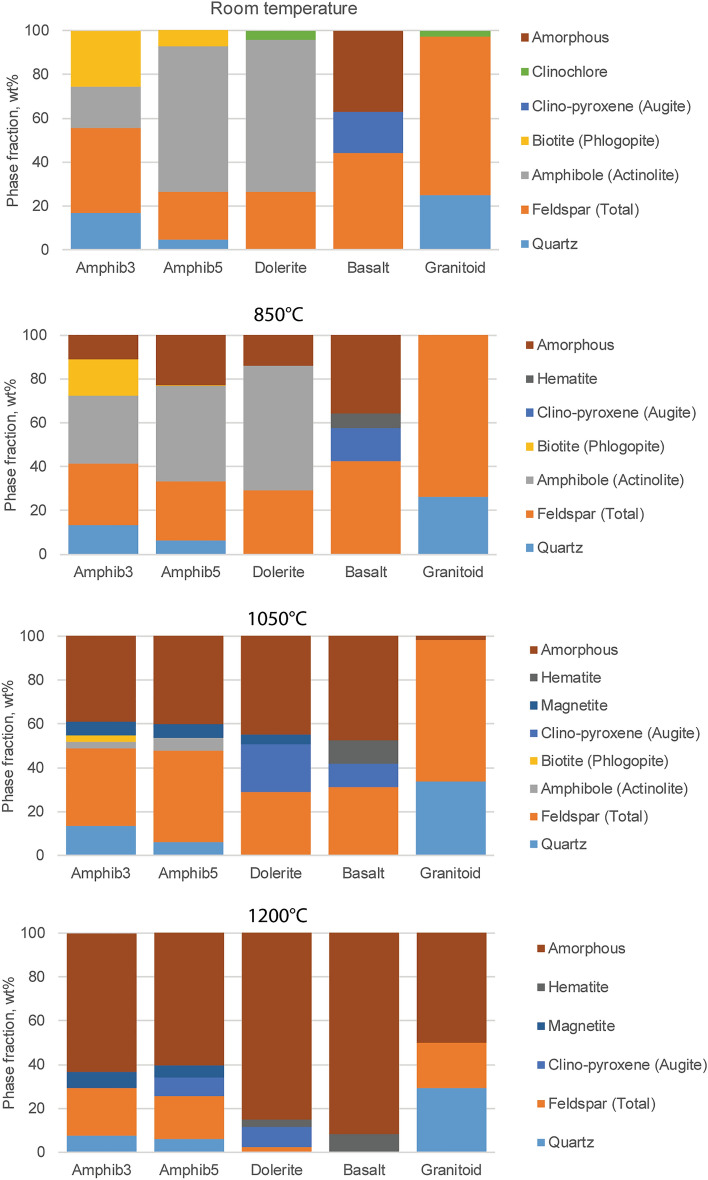
Ex situ XRD phase analysis of heated and quenched amphibolites (BA3, BA5), dolerite (D), basalt (U), and granitoid (RG), as a function of 15-min soak temperature: room temperature, 850 °C, 1050 °C, 1200 °C.

Figure 4 displays the phase assemblages versus temperature for amphibolite (BA3, BA5), dolerite (D), basalt (U), and granitoid (RG) rock samples, after isothermal holds at specified temperatures followed by air-quenching. Data for other samples is shown in Section S3. In all rocks except the granitoid, some melting starts by 850 °C, although in the basalt, original glass and new melt-quenched glass are indistinguishable. Evidently, up to 850 °C, the melt, which forms a glass upon quenching, results from the breakdown of hydroxyl and fluorine-containing minerals like micas (e.g., phlogopite, biotite) and chlorites (e.g., clinochlore), consistent with thermal analysis. By 1050 °C, water-containing amphibole minerals (e.g., actinolite, hornblende) have largely melted, as well as a small amount of feldspar. By 1200 °C, all rocks have melted significantly, to 50–90 wt%, after 15 min.

**Figure 5:**
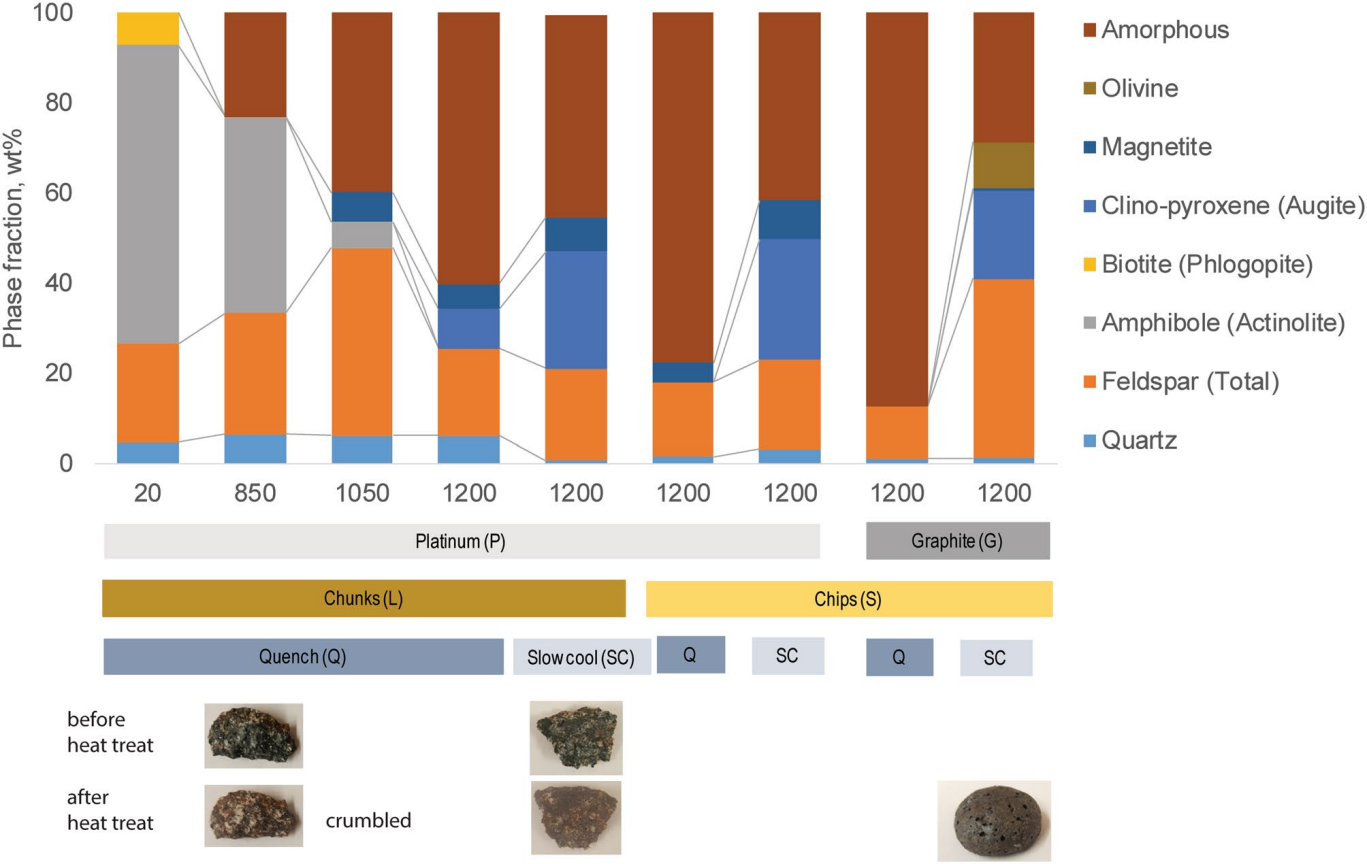
Ex situ XRD quantitative phase analysis of heat-treated amphibolite rock (BA5). Separate samples were heated to peak temperatures—20 °C (room temperature), 850 °C, 1050 °C, 1200 °C—and soaked for 15 min. Different experimental conditions were: crucibles (Pt or graphite), particle sizes (chunk or chip), and cooling conditions (quench or slow cool). Some representative photos of the samples are shown. Lines show some important comparisons.

Figure 5 displays the phase assemblages for amphibolite BA5 subsamples heated to 1200 °C, in Pt or graphite crucibles, and subjected to either air-quenching or slow-cooling. Samples were either chunks (2–3 cm rocks) or chips (collection of < 3 mm particles). One experiment, performed on BA5 chunks, involved 120 min of peak heating (vs. 15 min for other experiments) followed by a slow cool, and it showed negligible differences in the final phase composition for the longer heating time. Other conditions being the same, larger starting particle sizes (chunks of rock) produced less melt (glass), retained more quartz, and tended to produce some pyroxene upon quenching. By contrast, smaller particle sizes (chips) exhibited no pyroxene when quenched, whether heated in Pt or graphite, but rather exhibited a significant amorphous content (~ 75–90 wt%), with < 2 wt% quartz remaining, < 1 wt% magnetite created, and the balance of plagioclase feldspar. However, upon slow cooling, the amorphous fraction decreased drastically for both larger and smaller particles to 30–45 wt%, with larger fractions of feldspar being created in addition to clinopyroxene. Clearly, the formation of pyroxene takes time, and the greater thermal gradients between the centers of chunks and their surfaces slows cooling enough to allow substantial crystallization even under quench conditions. Graphite crucible experiments produced ~ 10 wt% olivine, demonstrating the necessity of a reducing environment to produce enough iron as Fe^2^^+^ to induce olivine formation. Clinopyroxene, olivine, and new feldspar—here fit as albite + microcline—all require slow cooling to form from the melt. It has been shown in various studies of vitrified hillforts that compositions of newly formed feldspar are typically non-equilibrium, having Na–K-Ca compositions in the thermodynamically forbidden region of the feldspar ternary phase diagram^40^.

**Figure 6:**
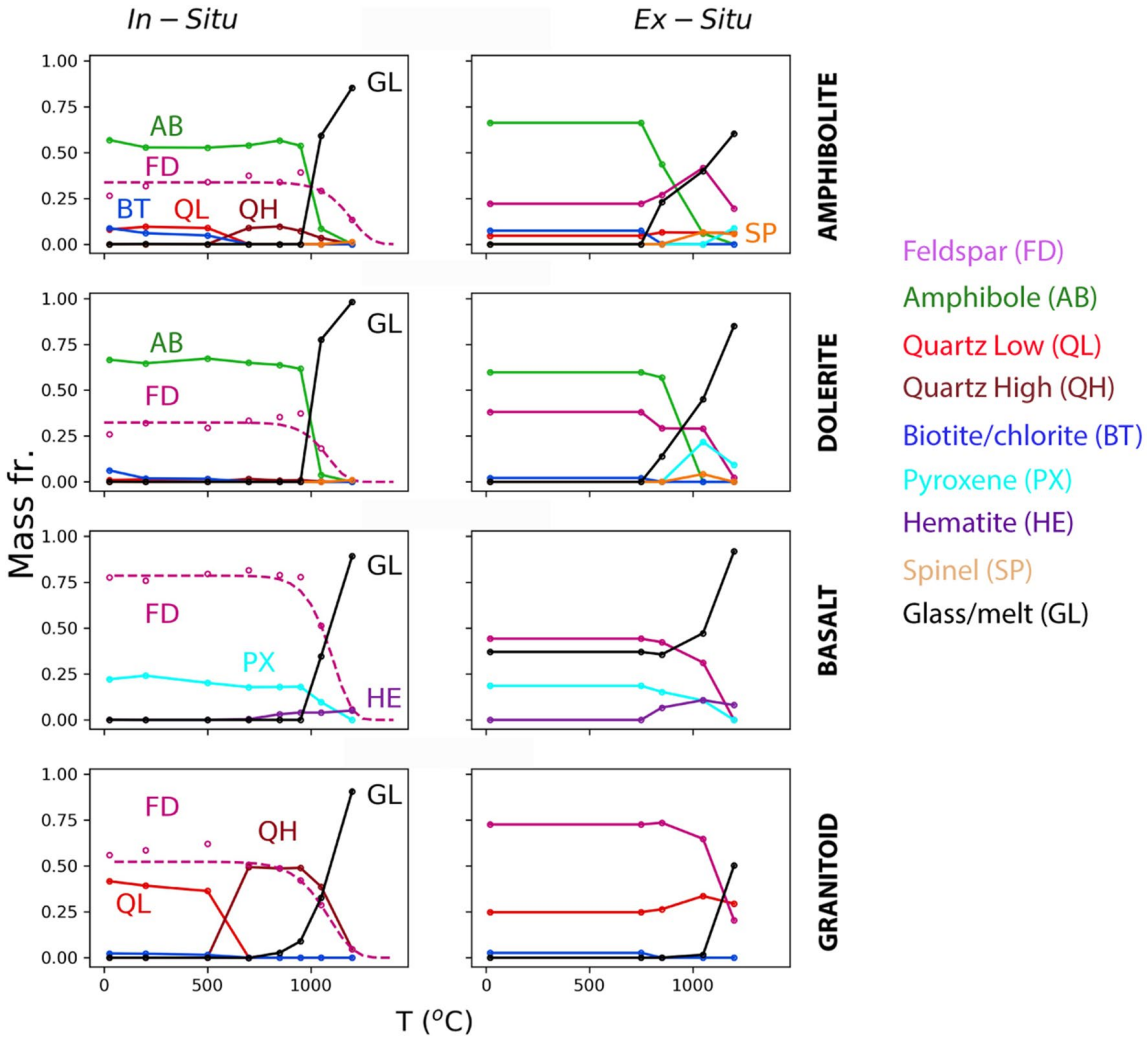
Phase analysis of heated rocks: amphibolite (BA5), dolerite (dike), basalt (HAS37), and granitoid (RG). Left column: In situ XRD with quantification by Rietveld refinement and calibration of the feldspar peak to determine amorphous fraction (see “Methods”). Right column: Ex situ XRD with Rietveld analysis; samples heated from room temperature to specified temperature at 10 °C min^−1^ as chunks in Pt crucibles, held for 15 min, then air-quenched and measured. Minerals shown: feldspar (FD), amphibole (AB), quartz low (QL), quartz high (QH), biotite + chlorite (BT), pyroxene (PX), hematite (HE), magnetite/spinel (SP), amorphous glass/melt (GL).

Figure 6 shows a comparison of the phase assemblages obtained by ex situ XRD and in situ XRD measured on heating, for amphibolite, dolerite, basalt, and granitoid, accounting for fits to the peak area of anorthite (see “Methods” and Section S4). Note that the temperature axis for the ex situ XRD is the peak temperature of the heat treatment for 15 min, while the temperature axis for the in situ XRD is the temperature at which the XRD pattern was measured. The results of the two methods are very similar, despite the fact that in situ XRD used rock powders due to requirement for good contact with the Pt heater strip, while ex situ XRD heat treatments used 2–3 cm chunks.

##### Amphibolite (BA5)

The amphibole fraction decreases sharply in the 850–1050 °C range, going to zero at 1200 °C. Similarly, the feldspar fraction drops at 1000 °C in the in situ experiment, but is still present in the ex situ data since new feldspar forms upon cooling. In the in situ experiment, biotite and chlorite breakdown is evident, and these phases are consumed between 500 °C and 700 °C. Moreover, quartz undergoes its low (α) to high (β) phase transition between 500 °C and 700 °C, which is not seen in the ex situ data since the quartz reverts to the α form. A small amount of spinel (i.e., magnetite) is seen at the highest temperatures in the in situ experiment, with much more seen in ex situ, which is consistent with magnetite predominantly forming upon cooling. Similarly, no pyroxene is observed in situ, but is observed ex situ, since it does form during cooling. This result is confirmed in the in situ XRD cooling measurements (Fig. S21), where magnetite forms at 1100 °C, and pyroxene and hematite form at 1000 °C. In the MELTS simulations (Fig. S22), which are equilibrium calculations, magnetite forms below 1400 °C at the hematite–magnetite (Hm–Mt) buffer, but only below 1200 °C for the fayalite-magnetite-quartz (FMQ) buffer. Olivine only forms in FMQ conditions, below 1200 °C, confirming the requirement of reducing conditions for olivine formation, as found in the ex situ experiments.

##### Dolerite (dike)

The dolerite behaves similarly to the amphibolite. A melt forms on heating between 950 °C and 1050 °C in situ, while some glass forms at lower temperatures, between 750 °C and 850 °C, in the ex situ experiment. High temperature phases include minor spinel in the in situ case at 1200 °C, where all feldspar and amphibole have been destroyed. In the ex situ case, however, spinel forms in the sample heated to 1050 °C—likely upon cooling—but the sample heated to 1200 °C forms hematite instead. Pyroxene only forms in the ex situ case, upon cooling. In the in situ cooling experiments (Fig. S21), both magnetite and pyroxene form below 1100 °C, with pyroxene the more dominant phase, until the temperature drops to 800 °C, when magnetite becomes dominant. In the MELTS cooling simulations (Fig. S23), magnetite is the liquidus phase below 1400 °C for the Hm-Mt buffer, and hematite is predicted to form below 1000 °C. Olivine is predicted to be the liquidus phase for the FMQ buffer, while feldspar and pyroxene crystallize below 1200 °C for both buffers. In no case, however, is feldspar observed in the in situ cooling experiments of melted rocks, despite its predicted crystallization from equilibrium thermodynamics. This can be attributed to the well-known issue regarding difficult homogeneous nucleation of feldspar in silicate melts^48^.

##### Basalt

Since this rock initially contains glassy material, some of the melt created at high temperature is a re-melting of the glass. In the in situ experiment above 950 °C, the crystalline phases can be seen to start melting with the decomposition of feldspar and pyroxene. Additionally, the hematite fraction grows above 700 °C, in both the in situ and ex situ experiments. The shape of the temperature dependence curve is also the same for each phase, but because the in situ experiment cannot distinguish between initial glass and melt-formed glass, the absolute quantities differ in the two experiments. In the in situ cooling experiment, when starting from complete melt at 1450 °C (Fig. S21), hematite and magnetite form below 1250 °C, with the bulk being magnetite. MELTS simulations (Fig. S24), show that for Hm-Mt (FMQ) buffer, magnetite should form below the 1300 °C (1400 °C) liquidus, and feldspar and pyroxene should form below 1200 °C (1300 °C). While at equilibrium below 1200 °C, hematite forms under Hm-Mt buffer conditions rather than pyroxene. Thus, the cooling experiments indicate that a moderately oxidizing environment creates hematite but not pyroxene, the latter being the preferred phase at equilibrium in a more reducing environment. As described above, feldspar is predicted but not observed. When cooled under oxidizing conditions, the amount of pyroxene predicted for this basalt composition is quite low compared to the amphibolite and dolerite seen above.

##### Granitoid (RG)

The granitoid sample here is (wt.%) 47% plagioclase, 26% K-feldspar, 25% quartz and 3% chlorite at room temperature. For both in situ and ex situ heated samples, melting begins near 1000 °C, with a slightly lower temperature observed for the in situ sample. Near complete melting is observed by 1200 °C for the in situ sample. As with the amphibolite, a transition from low to high quartz is observed near 500 °C, though only low quartz is seen in the room temperature ex situ sample, due to reconversion upon cooling.

#### Chemical and mineralogical textures

##### Heat treated amphibolites

**Figure 7:**
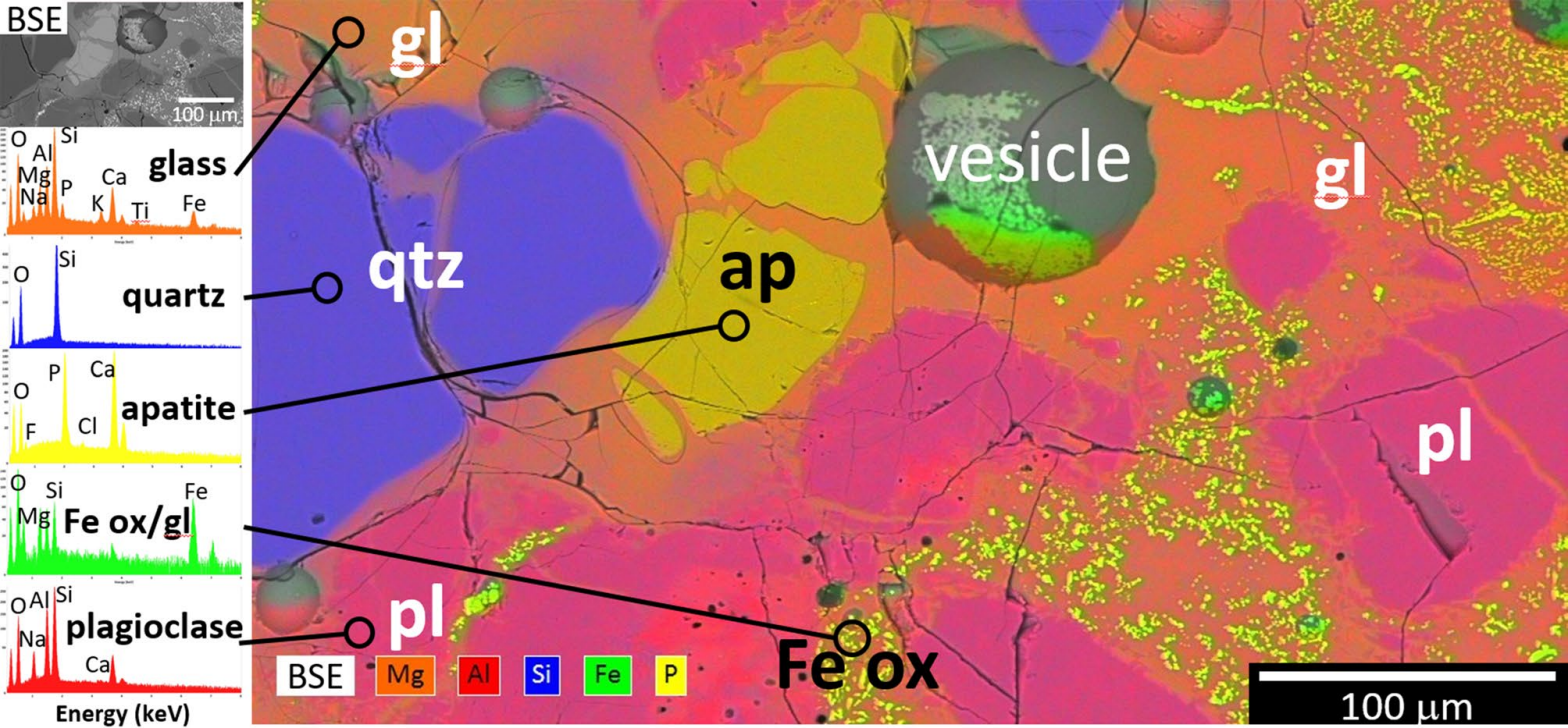
Microstructure and chemistry of heated amphibolite BA3 (1200-P-L-Q condition: rock chunk heated to 1200 °C in Pt, held 15 min then quenched in air). SEM-BSE image, superimposed false color X-ray map, and spectra for each chemical phase derived from the image (Al-red; Fe-green; Si-blue; P-yellow; Mg-orange). Micrometer-scale Fe oxides precipitated from the melt while all other solids represent incompletely melted minerals. Phase fractions determined by XRD (wt%): 63% glass, 2% feldspars, 8% quartz, 7% magnetite.

**Figure 8:**
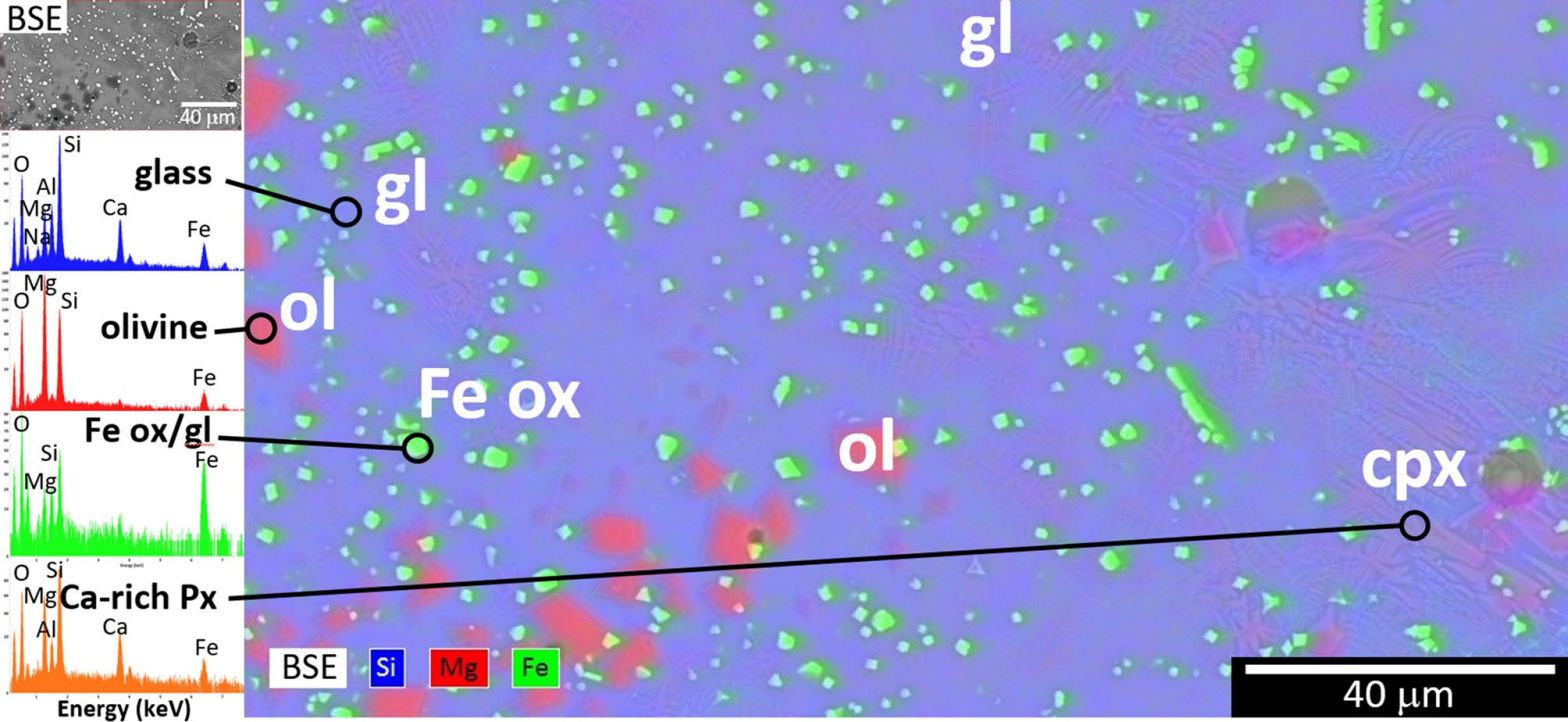
Microstructure and chemistry of heat-treated amphibolite BA5 (1200-P-L-Q condition: rock chunk heated to 1200 °C in Pt, held 15 min then quenched in air). SEM-BSE image and SEM–EDS map, colored according to phases with shown X-ray fluorescence spectra. The liquidus phases precipitating from the glass were a Fe–Mg–Al spinel and Mg-rich olivine. Clinopyroxene (augite) quench crystals were also observed. Different phases were seen in other regions, including quartz and feldspar.

Figure 7 shows a representative microstructure from one of the melted amphibolites (BA3). Other images are shown in Figs. S25–S31. For the short heat-treatment time (15 min), one sees relict quartz and apatite, as well as slightly altered plagioclase feldspar. Presumably the chemical non-uniformity observed is the result of incipient melting during heat treatment. Numerous small Fe oxide crystals, containing Mg, Al, and Fe, are present in regions of glass created by the melting of quartz, feldspar, and amphibole. Figure 8 shows a representative microstructure from another of the melted amphibolites (BA5), with further images in Figs. S32–S39. Similar to BA3 above, heat treated under the same conditions, residual quartz and feldspar are noted. Some olivine rich in Mg is present, though the quantity of this phase is below the detection limit for XRD. Ca-rich pyroxene (augite) forms as quenched dendrites, along with Fe oxide.

##### Heat treated granitoids

Figure S40 shows representative SEM-BSE images of heat-treated granitoids (white gneiss—WG, and red gneiss—RG) at 1050 and 1200 °C, held for 15 min followed by air quenching. Upon heat-treatment at both temperatures, the WG converts to a large fraction of glass containing small amounts of spinel and quartz. However, the RG forms barely any glass upon heat-treating at 1050 °C, and contains large amount of quartz with alkali feldspars and some mica. These results are broadly consistent with the XRD. Both RG and WG become mostly glass after heat-treatment at 1200 °C. Chemically, this glass is mostly low-Fe “clear glass”—darker in BSE owing to lower average atomic number—with some high-Fe “dark glass”—brighter in BSE. The glass compositions were measured using EPMA point scans, and their mean compositions averaged from 5 to 10 individual scans are listed in Table 1.

**Table 1:** Elemental composition (wt% oxides) of various glasses measured in the heated amphibolites (BA3, BA5) and granitoids (WG, RG), as well as an archaeological sample (Bro-W).

Sample	Phase		Composition, wt% oxides										
			SiO_2_	Al_2_O_3_	Na_2_O	MnO	FeO_t_	CaO	K_2_O	TiO_2_	MgO	P_2_O_5_	Total
BA3-1200-P-L-Q	Dark glass	Avg	48.34	14.25	1.96	0.20	10.52	12.87	1.72	0.73	6.32	3.09	100.00
		St. Dv	3.66	2.12	0.27	0.09	1.51	1.94	0.31	0.23	0.58	1.69	–
BA5-1200-P-L-Q	Dark glass	Avg	53.46	10.44	1.39	0.25	12.19	12.46	0.44	0.91	8.45	b.d.l	100.00
		St. Dv	1.36	0.33	0.13	0.08	0.91	1.20	0.08	0.20	0.97	–	–
WG-1200-P-L-Q	Clear glass	Avg	61.25	21.65	6.03	b.d.l	0.20	1.25	6.47	b.d.l	0.05	b.d.l	96.61
		St. Dv	3.84	4.23	0.72	b.d.l	0.11	0.98	1.47	b.d.l	0.03	b.d.l	–
	Dark glass	Avg	51.88	15.63	4.34	0.49	9.14	5.48	2.80	1.64	4.19	0.79	96.37
		St. Dv	1.06	2.49	1.39	0.07	1.63	1.54	0.47	0.61	0.34	0.17	0.86
RG-1200-P-L-Q	Clear glass	Avg	65.36	19.60	6.93	b.d.l	0.22	0.90	4.86	b.d.l	b.d.l	b.d.l	97.87
		St. Dv	1.58	0.47	1.26	b.d.l	0.18	0.43	2.52	b.d.l	b.d.l	b.d.l	–
	Dark glass	Avg	56.11	17.53	4.17	0.07	2.90	10.86	4.06	0.99	0.41	0.23	97.35
		St. Dv	1.88	1.61	2.41	0.03	1.41	3.62	2.50	2.13	0.49	0.47	–
Bro-W (trace 1)	Clear glass	Avg	66.46	19.07	3.09	0.03	1.02	1.22	9.11	b.d.l	0.33	b.d.l	100.33
	Dark glass	Avg	49.63	17.34	2.63	0.42	12.36	5.96	3.15	2.42	5.01	0.65	99.58
Bro-W (trace 2)	Clear glass	Avg	66.86	19.67	3.67	0.04	1.33	1.10	7.51	b.d.l	0.41	b.d.l	100.60
	Dark glass	Avg	54.88	17.10	3.06	0.34	10.74	4.36	2.82	1.97	4.41	0.36	100.03
Kresten^7^ (Broborg)	Clear glass	Avg	61.7	22.1	5.8	0.0	1.0	1.4	5.9	0.1	0.3	0.2	98.5
	Dark glass	Avg	51.1	16.5	2.8	0.3	9.39	9.8	1.3	0.85	6.2	0.3	98.54
Weaver^32^ (Broborg)	Clear glass	Avg	67.37	15.12	4.71	0.02	0.82	0.22	8.94	0.15	1.18	1.41	99.94
	Dark glass	Avg	48.95	14.19	4.76	0.34	10.57	11.33	2.39	1.63	2.69	1.98	98.83
Lambert^49^ (CRB)	Clear glass	Avg	74.40	13.00	2.60	n.m	1.60	0.50	6.60	0.90	0.30	0.40	100.30
	Dark glass	Avg	52.40	5.60	0.00	n.m	29.40	2.30	0.50	0.00	9.10	0.00	99.30

n.m. indicates not measured; b.d.l. indicates below detection limits.

For the archaeological samples, the reported values are those at the ends of the trace at the interface between dark and clear glass (see Table S2). Fe oxide for all shown as FeO_t_, with no assumption about oxidation state. For interpretation of sample names, refer to “Methods” section.

## Discussion and implications

### Glass compositions

**Figure 9:**
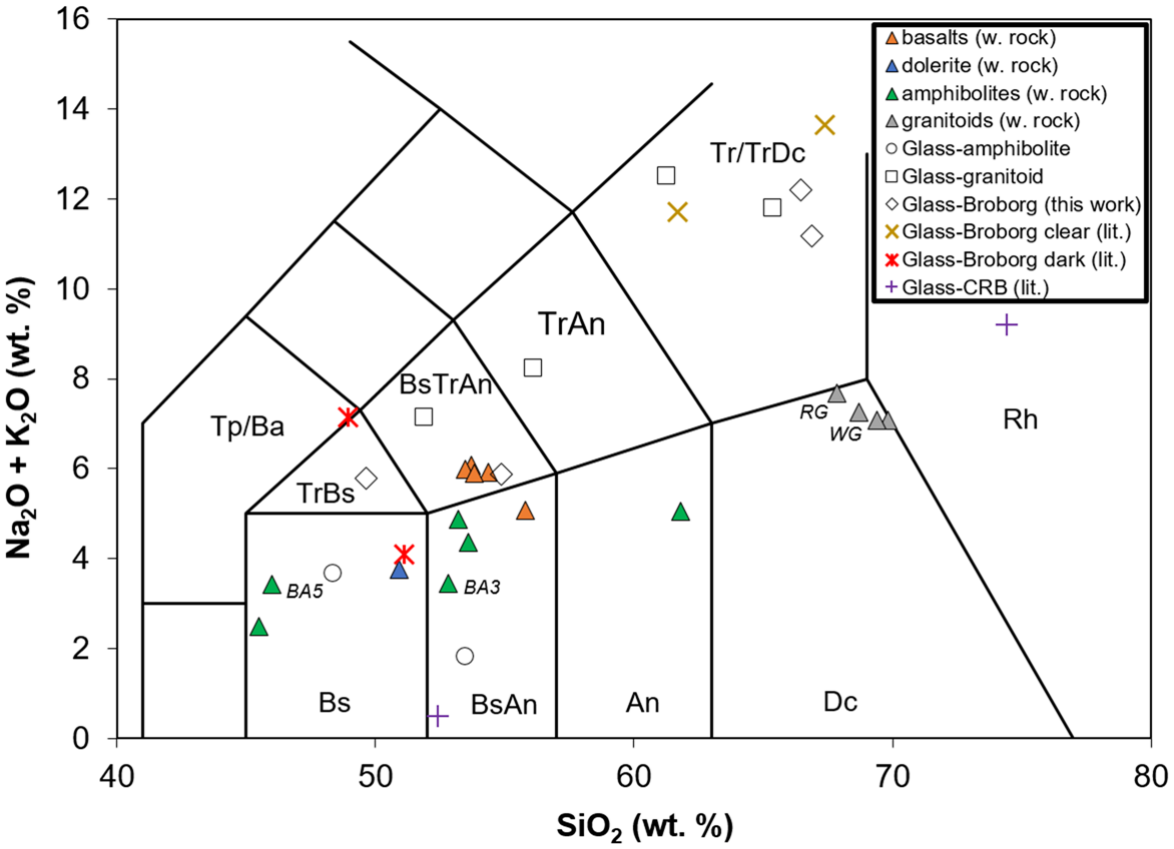
Compositional space of considered whole rocks (triangles) including basalts, dolerite, granitoids, and amphibolites, several of which are labeled, as well as measured glass compositions from this work (open symbols) and literature (other symbols) as shown in Table 1. Diagram based on the total alkali silica (TAS) diagram of Le Maitre et al.^61^ for volcanic rocks. Regions of TAS relevant to the data are: Basalt (Bs), Basaltic Andesite (BsAn), Andesite (An), Dacite (Dc), Trachyte/Trachydacite (Tr/TrDc), Trachyandesite (TrAn), Basaltic Trachyandesite (BsTrAn), Trachybasalt (TrBs), Tephrite/Basanite (Tp/Ba).

A summary of the glass compositions considered in this work is shown in Table 1 and summarized on the total alkali-silica (TAS) diagram (Na_2_O + K_2_O versus SiO_2_ wt%) in Fig. 9. Also plotted on the TAS are the starting rock compositions, with their corresponding full compositions shown in Table 2 and Table S3. Amphibolites, dolerites, and basalts have basic to intermediate compositions in the regions described as: basalt, basaltic andesite, and basaltic trachyandesite. Melts measured from the amphibolites lie in these aforementioned regions. The granitoids from Broborg lie in the corner of the dacite region near rhyolite and trachydacite. The basalt compositions lie in the basaltic andesite region.

**Table 2:** Summary of ex situ melting experiments.

Test	Rock	Crucible	Samples	T_max_ (°C)	Dwell (min)	Cooling	Notes
BA1-*T*-P-L-Q (or SC)	BA1	Pt	Chunks	T = 850, 1050, 1200*	15	Quench (or *slow cool)	See supplemental
BA2-*T*-P-L-Q (or SC)	BA2	Pt	Chunks	T = 850, 1050, 1200*	15	Quench (or *slow cool)	See supplemental
BA3-*T*-P-L-Q (or SC)	BA3	Pt	Chunks	T = 850, 1050, 1200*	15	Quench (or *slow cool)	1200 Q and SC measured
BA4-*T*-P-L-Q (or SC)	BA4	Pt	Chunks	T = 850, 1050, 1200*	15	Quench (or *slow cool)	See supplemental
BA5-*T*-P-L-Q (or SC)	BA5	Pt	Chunks	T = 850, 1050, 1200*	15	Quench (or *slow cool)	1200 Q and SC measured
BA5-1200-P-L-SC-120	BA5	Pt	Chunks	1200	120	Slow cool	Longer dwell
BA5-1200-P-S-Q	BA5	Pt	Chips	1200	15	Quench	
BA5-1200-P-S-SC	BA5	Pt	Chips	1200	15	Slow cool	
BA5-1200-G-S-Q	BA5	Graphite	Chips	1200	15	Quench	
BA5-1200-G-S-SC	BA5	Graphite	Chips	1200	15	Slow cool	
BA5-1200-G-S-SC-clay	BA5	Graphite	Chips	1200	15	Slow cool	50 wt% Broborg clay
BA3-1200-P-S-SC-gran	BA3	Pt	Chips	1200	15	Slow cool	50 wt% #433 granite
D-*T*-P-L-Q	Dike	Pt	Chunks	T = 850, 1050, 1200	15	Quench	
UB-*T*-P-S-Q	UMAT	Pt	Powder	T = 850, 1050, 1200	15	Quench	
RG-*T*-P-L-Q	RG	Pt	Chunks	1050, 1200	15	Quench	
WG-*T*-P-L-Q	WG	Pt	Chunks	1050, 1200	15	Quench	

For the particle size, ‘chunks’ are listed as L (large) and ‘chips’ are listed as S (small). Sample name is Rock-Temperature-Crucible-Particle Size-Cooling Condition.

The nominal glass composition for heat treated amphibolites is basaltic. Some pockets of basaltic glass in the midst of precipitated spinel crystals are slightly depleted in Fe, but enriched in Al and Ti. Melted granitoids produce large amounts of clear (felsic) glass and small amounts of dark (mafic) glass. The clear glass is a trachyte and the dark glass is trachyandesite (i.e., basaltic). These cannot be predicted from the whole rock chemistry of the granitoids, which upon equilibrium melting and quenching would yield glasses in the rhyolite or dacite region—as confirmed by MELTS calculations. *The only way to obtain clear glass compositions in the trachyte region is by nonequilibrium melting of feldspar with or without some quartz.* The mafic glass measured for the melted granitoids correspond to regions of melted mafic minerals such as biotite, which are too minor to be picked up by XRD, but which are easily seen in hand specimens, thin section, or electron microscopy. Columbia River and other basalts have also been observed to have natural regions of immiscible rhyolite clear glass and basalt/basaltic andesite dark glass components^49,50^. The clear glasses observed in basalts have slightly higher Si and lower Na and K than measured for Broborg, while the dark glasses in phase-separated basalt are lower in Al and Na and higher in Fe and Mg than those for Broborg (see Table 1).

The Broborg vitrified wall samples show evidence of dark and clear glass in contact (Fig. S4 and Table S2). The endmembers of two-line scans across this interface show dark glasses as trachybasalt or basaltic trachyandesite, and clear glasses as trachyte. Finally, these compositions compare well to measured Broborg hillfort glasses from Kresten et al.^21^ and Weaver et al.^32^, with clear glass compositions in the trachyte region and dark glass in trachybasalt or basalt regions. Phase-separated basalt glasses from Lambert et al.^49^ differ in being rhyolitic for the clear glass and basaltic andesite with very low alkali for the dark glass.

These results suggest that the origin of the clear glass at Broborg is partial melting of the granitoid rocks, leading to compositions in the trachyte region of the TAS diagram, with high silica and high alkali. The dark glass is most likely due to melted amphibolite, though melts of mafic minerals within granitoids make similar glass compositions with somewhat higher alkali content. The dark glasses have SiO_2_ < 55 wt%, with total alkali < 9 wt% and tend to have significant Fe (average total Fe as FeO (FeO_t_) ~ 9 wt%) and higher Ca, Mg, and Ti than the clear glasses. Alumina concentration is high in both glasses—averaging 15–20 wt%—being higher on average in the clear glasses, consistent with a dominant contribution from feldspar. The average measured dark glass has a composition at the corner of the basalt-trachybasalt-basaltic andesite regions and on the border between basic and intermediate rocks according to silica content. It is evident from these measurements, as well as the samples’ microstructures, that *most measured glassy regions in vitrified hillforts cannot be predicted based on overall rock compositions*, since rarely are rocks completely melted.

### Crystalline phase development

The melting conditions at Broborg appear to have been quite nonequilibrium and occurred under conditions uncommon for geological crustal or mantle melting. The conditions were at atmospheric pressure but high temperature for short times—minutes to weeks, at most. For this reason, some phases that were predicted to form at equilibrium either may not have formed at all (i.e., feldspar in the in situ cooling XRD) or only formed upon slow cooling (i.e., pyroxene in the amphibolite melts). Microstructures and chemistries similar to those observed in the Broborg vitrified wall samples were obtained in this work. For the amphibolites, the breakdown of biotite above ~ 850 °C and amphibole above ~ 1000 °C results in melts which then quench to mafic glass, or crystallize slowly to contain pyroxene + spinel (~ magnetite) + olivine (under reducing conditions) + feldspar. We know that a reducing atmosphere and a slow cooling rate are required to produce the olivine seen in the archaeological samples. These melting and cooling reactions in the amphibolite rocks are much more complicated than those reported for hillforts that involve only sandstones or granites^20^.

We have not, however, been able to reproduce the cordierite crystalline phase seen in the archaeological sample Bro-B. Experiments attempting to recreate this phase, where local clay was added to increase the Al content, were unsuccessful. It has been suggested that clay may have been added during the construction, particularly to reinforce firing holes (Fig. 1f) during use, areas in the wall where charcoal may have been repeatedly added to keep the temperatures high^6,34^. However, in our experiments with added clay, no additional crystalline phases could be produced upon slow cooling, and only feldspar and quartz were observed. Thus, we cannot yet ascertain the formation mechanism of cordierite, but as previously stated, it is common in geological low-pressure, high-temperature and pyrometamorphic systems^39^. Cordierite has been observed as a phase in Bernstorf hillfort in Germany^51^, and the authors attribute its formation to the locally higher temperatures achieved in the center of the rampart, of ~ 1200 °C^9^.

### Factors for interpreting the archaeology

If the wall vitrification at Broborg was deliberate, as has been suggested^21^, it is likely that local Iron Age people could have managed it. The pyrotechnology was widely available from long experience with iron-smelting in the region, and temperatures of 1000–1200 °C were routinely achieved (e.g., drafted bloomery iron smelting furnaces ~ 1250 °C; iron smithing hearths ~ 800–1100 °C; copper and alloy casting hearths ~ 900–1200 °C)^52^. However, as has been demonstrated by many attempts at large scale reproduction—discussed in the introduction—heat management is not trivial. At Broborg, there is evidence of depressions on either side of each wall section interpreted as firing holes (Fig. 1f) described in detail and interpreted at Broborg here for the first time^34^. In the excavation^34^, the region on the inside of the wall downslope from the firing hole showed ash, interpreted as wood or charcoal which had burned and was swept out to provide room for fresh charcoal and air intake for the bellows. These holes are commonly observed features in other extensively vitrified hillforts in Scotland^5^ and France^6^, but they have been variously interpreted.

For a given temperature above ~ 1000 °C, the amphibolite would have melted enough to drip over time (Fig. 1n) and leave impressions of wood or charcoal (Fig. 1k), while the granitoid at these temperatures has a higher viscosity by about 2 orders of magnitude and often results in only a superficial clear glass (Fig. 1m). It may be that the higher temperatures required for granitoid softening resulted in the walls maintaining their shape, while the more fluid amphibolite melt—or other mafic minerals from the granitoid—provided the “cement” to join the rocks (Fig. 1h). The granitoid material shows macroscopic changes which depend on the maximum temperature achieved. When heated between 500 and 1000 °C, mica changes from black to brown due to the oxidation of iron, and defines the 500 °C minimum isotherm of golden biotite (Fig. 1i)^29^. Above 1000 °C, the mica minerals melt^29^, and the rocks are observed to weep a dark melt, often along lines, while more refractory felsic minerals remain intact (Fig. 1j). At the highest temperatures, seen in the center of the wall (see Fig. 1g), even the felsic minerals of the granitoids are sufficiently hot to produce a massive white and black melted material (Fig. 1l).

### Implications for vitrified hillforts in general

We have also shown that the fort vitrification process, in general, is rather insensitive to rock type, provided that the temperatures are above ~ 1000 °C (where some melting is expected) or above ~ 1200 °C (where extensive melting is expected even in short times). Heating times were likely much greater than the 15 min studied here, so it is quite likely that extensive melting (i.e., above the solidus) would have taken place regardless of lithology. Thus, while lithology may have affected the *feasibility* of extensive vitrification, as suggested by Wadsworth et al.^20^, the evidence that humans *selected* rocks for their lower temperature melting characteristics, as suggested by Kresten et al.^21^, is harder to corroborate here from just the melting temperatures. Rather, as noted by Nisbet^26^ from empirical observation, vitrification in hillforts has been observed for many different source rocks and many lithologies throughout the world. Perhaps the most convincing evidence of this is the co-occurrence of vitrified (i.e., heated siliceous rock) with calcined (i.e., heated calcareous rock) material at Begues in France^5,6^. Evidently, temperatures ~ 1000 °C are sufficient for at least superficial melting in many rocks.

Various factors have been speculated to further reduce melting points and thus facilitate the creation of the vitrified walls. For example, higher Fe^2+^ content, such as might be achieved in a reducing fire, could lower melting temperatures, and addition of water vapor might also reduce melt viscosity. Additionally, various experiments over the years have looked at additions of other materials which might lower the melting point of the rock (i.e., alkali ash from wood, salt, clay, or bone)^6^, but none has been found to have a profound effect. These additives are not required to achieve melting, since it has been shown that the required temperatures to produce some melts are, in fact, modest and comparable to those from contemporaneous pyrotechnologies.

However, it is still useful to compare the different lithologies in terms of how easy it might have been to obtain extensive pockets of melt. By inspection of Fig. 4, it can be seen that the most extensive melting occurs for basalt, possibly due at least partially to its pre-existing amorphous phase. Furthermore, the other compositions of amphibolite and dolerite all behave similarly. Several other amphibolites were also tested and while not discussed in detail, the data is shown in Table S5. Here it was found that rocks with lower quartz content and higher amphibole content melted more extensively for a given set of thermal conditions. Moreover, all of these melted more readily than the granitoid material. It was also noted that a granitoid material (WG) which had apparently been pre-heated somewhat—as evidenced from its microstructure and quench crystallization—produced a larger quantity of glassy material at a lower temperature than the pristine granitoid (Table S9). Thus, it is possible that repeated heating attempts would have eventually created enough melt for the rocks to fuse, so that a lower temperature could be used after several attempts. Proof of preference for pre-heated rocks, or those with low quartz and high amphibole content, if found, would suggest deliberate choices towards construction.

The heat content of the fuel is also important for achieving the high temperatures needed for vitrification^6,53^, particularly when parasitic cooling (e.g., cold wind, rain) also occurs^1^. Charcoal is an obvious choice for a higher quality fuel, and charcoal production could have easily been accomplished offsite and transported to the hillfort or collected after a major fire. Other potential fuel sources, though none were known to be used for iron smelting, include bone marrow^54^ (bones have been found in some vitrified hillforts^6^) and tar^55^. Though tar may not have been used directly, charcoal produced as a byproduct of its beneficiation might have been. Recent archaeological evidence shows tar production pits in the vicinity of Uppsala, dating from c. 100–400 CE^55^, around and slightly before the dating for the vitrification at Broborg, c. 400–600 CE^33,34^.

Finally, recent excavation^34^ has shown evidence of human activity inside the hillfort which can be dated by stratigraphy as being after the vitrification of the wall. If the destruction of the walls was the purpose, it seems unlikely that there would be subsequent habitation of the site. Through a combination of C-14 dates, this habitation layer is 432–542 CE (middle Migration Period). This dating overlaps the best paleomagnetic dates for the vitrification event, which is 389–579 CE^33^. Therefore direct dating cannot establish this sequence, but stratigraphy suggests the habitation after the vitrification. The different analyses (osteology, macrofossil analyses and wood species analyses) have contributed to painting a picture of the activities in the fort during the Migration Period. Behind the walls, people may have cooked and consumed food in the form of meat, grain/bread and spices. The food would probably have been cooked in hearths, cooking pits or in low-temperature ovens. Foods may also have been cooked in protective clay and herb wrappings in direct contact with embers. Spices like juniper berries were probably collected in the immediate vicinity. The people present at Broborg also had access to oats, shell grain and emmer/spelt wheat. Meat consumption consisted of sheep/goat and beef and, probably, also pigs. Both conifers and deciduous trees were used as fuel.

The experimental results here, and those for other vitrified hillfort sites, suggest that vitrification in general occurs in most lithologies starting at temperatures above 1000 °C. Even short heating times (~ 15 min) with most rocks produces superficial melts, which could fuse together rock walls. This facility for melting makes interpretation of the archaeological and geological record more challenging, since vitrification could have come about at many sites for many different lithologies for many reasons. The extent of melting is strongly dependent upon particle size, and previous excavators have argued that fist-sized amphibolite rocks at Broborg were intentionally fabricated for building the wall^7^. The extent of the melting at Broborg and other similar sites does suggest the conditions for vitrification was different than at other sites. Perhaps more importantly, previous large scale testing has shown that heat management to obtain temperatures > 1000 °C is not trivial, but rather a significant engineering issue. The excavation stratigraphy suggests that occupation and human activity did occur after the vitrification event. Therefore, it should not be ruled out that clever Iron Age peoples both could have and did construct the inner walls at Broborg to facilitate vitrification. If the vitrification was deliberate and constructive, the discovery of the motivation for this expenditure of effort and ingenuity will necessitate further research and speculation about the minds of ancient engineers.

## Conclusions

In this study, we performed a series of experiments to attempt the recreation of conditions used to melt the stone walls in the Swedish vitrified hillfort of Broborg. We collected potential source rocks locally available in SE Sweden—granitoid, amphibolite, and dolerite—and assessed bulk chemistry, mineralogy, and melting behavior. We studied the mineralogical textures of rocks melted under various conditions and compared these to samples from the vitrified wall. Various melting parameters were tested, including the minimum and maximum melting temperatures, cooling rate, starting particle size, and presence of additional materials (i.e., clay). Ex situ and in situ X-ray diffraction was performed and compared to equilibrium crystallization calculations, and we analyzed selected experimental samples for major element distributions. Furthermore, we successfully reproduced most features that were observed archaeologically, including dark and light glasses and all crystalline phases except cordierite.

Results indicate that the reported dark glass at Broborg is formed from the melting of amphibolite or dolerite rocks at 1000–1200 °C. Slow cooling (~ 10 °C min^−1^) and reducing conditions are required to create the observed mineral assemblage, including glass, pyroxene, spinel, olivine, new feldspar, and minor relict quartz. Heating destroys the original volatile-containing minerals—mica at lower and amphibole at higher temperatures—incorporating the elements into the melt, and subsequently crystallizes the aforementioned new phases during slow cooling. At similar temperatures, melting of the granitoid rocks alone produce the reported clear glass as a non-equilibrium quenched trachyte composition melt, formed primarily from melted feldspar. Given the expected viscosities of these two compositions at 1200 °C, contact mixing is slight, and the observed two-glass behavior is predicted.

## Methods

### Rock selection

#### Archaeological hillfort samples

Two archeological samples from Broborg were obtained, as previously described^32^. These samples consisted of vitrified rock taken from the inner wall, some of which can clearly be seen as being composed of one type of rock (i.e., granitic-gneiss) fused by a black glass (i.e., melted amphibolite) (Fig. S1). The basic mineralogy resulting from melting of these starting materials had previously been established and discussed^7,21^. Prior to cutting these samples, X-ray tomographs (XRT) were obtained to assess suitable locations for sub-sampling, as described elsewhere^31,56^. Sub-sectioned samples of “PNNL-Broborg#15” (named Bro-B here for ‘black’) and a subsection of “PNNL-Broborg#215A” (named Bro-W for ‘white’) were thin sectioned and investigated by optical and electron microscopy. Remainder material was crushed for X-ray diffraction.

#### Source rocks (igneous and metamorphic)

The primary mafic rock thought to be responsible for the dark basaltic-composition glass at Hillfort has historically been amphibolite^21,32^. However, it had not been clear what the importance, if any, of the granitic gneiss component was to the melting event. Hence, a number of amphibolite and granitic/gneiss samples were collected at and near the Broborg site.

##### Amphibolite

Amphibolite samples were collected from five outcrops within 6 km of the site and one boulder on the site. The locations of these outcrops is described in detail in a report^57^. These samples were denoted BA1-BA5 and BB6. These rocks were broken fresh from the outcrops (BA1-BA5) or boulder (BB6). Categorization of the amphibolite mineral assemblage by XRD effectively separated them in the same categories obtained by bulk rock chemistry in the total-alkali-silica diagram (Na_2_O + K_2_O versus SiO_2_ wt%), assuming isochemical metamorphism from igneous rock protoliths^57^: BA2, andesite (~ 62 wt% SiO_2_); BA1, BA3, BA4, basaltic andesite (~ 53 wt% SiO_2_); BA5, BB6 and the dolerite sample, basalt (~ 45–51 wt% SiO_2_). These distinctions are important, as SiO_2_ and amphibole contents affect melting behavior.

##### Dolerite

During excavations in fall 2017, a dike of dolerite (i.e., diabase) was found running through the bedrock of the hill on which the fort was built, and samples were collected of this material. This rock had similar mineralogy and composition to the amphibolites, with higher amphibole and lower quartz.

##### Granite

Local felsic rocks at Broborg could be classified as granites with more or less metamorphosis to gneiss. Most of the local felsic rocks are from glacial till, but the Broborg hill itself appears to be composed of bedrock of a pink granodiorite. Thus the collected granites are not originally local since they have been transported by recurrent glaciation. Three main types of granitoids are present in the area: felsic granite, intermediate granite-granodiorite, and the basic granodiorite-tonalite. The felsic red granite mainly consists of plagioclase, K-feldspar, quartz and biotite (dark mica), while the intermediate red to gray varieties contain less K-feldspar but also amphibole. The basic gray granitoids are poor in K-feldspar, rich in plagioclase and generally also in amphibole, and often contain dark mafic enclaves showing magma mingling. The granitic varieties are generally reddish to pink due to abundant K-feldspar, while the granodiorites are grayer. The two types often appear together with diffuse contacts. Several hand specimens were collected of visually distinct material from the surface (i.e., not hammered from outcrops or boulders). The ones considered in detail are denoted RG (red granitoid) and WG (white granitoid), though others were also investigated and are described in the Section S2.

##### Basalt

For comparison with other hillfort lithologies, and to assess the melting differences in rocks with mafic composition, a set of Columbia River Basalts containing matrix glass was collected and assessed as well, including those from the Umatilla member of the Saddle Mountains Basalt (UMAT-1, HAS37) and the Grande Ronde Basalt. Details of these rocks have been previously published^58,59^.

### Heat treatment of source rocks

Samples were subjected to various heat treatments for analysis of the development of phases as a function of processing parameters (i.e., isothermal heat treatments, effect of sample volume, and effect of reducing or neutral atmospheres). The characterization plan is described after.

First, heat treatments were performed using monolithic samples (referred to as “chunk” or “monolith” samples) with dimensions of 2–3 cm. Data has been omitted from some of the amphibolites considered in Ogenhall^57^ because of the difficultly of manually separating quartz-rich seams from the surrounding rock. Chunked rock pieces were placed in 1.5 × 1.5 × 1.5 in^3^ unlidded platinum containers and inserted into an electric furnace (Deltech drop-bottom) pre-heated to 850 °C, 1050 °C, and/or 1200 °C, held for 15 min, then removed and *air-quenched* (cooling rate estimated^60^ at 10–100 °C s^−1^). This protocol was conducted on amphibolites—reported here are BA3 and BA5 as representative—the dolerite (dike), a basalt (UMAT), and two granite samples (white and red gneiss). Data on other samples are provided in the supplementary.

Second, heat treatments were performed using similar monolithic samples, which were heated on Pt foil in an electric furnace (Sentrotech) from ambient at ~ 6 °C min^−1^ to 1200 °C, held for 15 min, and then *slow-cooled* from the designated temperature to room temperature at a rate of ~ 10 °C min^−1^. Note that in practice the furnace does not dissipate heat quickly enough to cool at this rate, so 10 °C min^−1^ is the maximum cooling rate. One test was conducted with a longer hold time at temperature of 120 min for comparison. These tests were performed only on the amphibolite samples.

Third, the quench and slow-cooling heat treatments on BA5 amphibolite were repeated using small *chips* (estimated to be < 3 mm) of amphibolite placed in Pt or graphite crucibles, heated at 10 °C min^−1^ to 1200 °C, and either air quenched or slowly cooled (i.e., cooled at 10 °C min^−1^ (~ 0.2 °C s^−1^) as above). The purpose of these experiments was to test the effect of redox conditions on melting and crystallization. Fine chips were used so the rock would be more uniformly exposed to the reducing graphite crucible.

To test the hypothesis that clay may have been added purposefully^6^ or inadvertently to the hillfort before firing, one test was conducted where 50 wt% of dry clay—collected in a crop field at the foot of the hill at the Broborg site—was mixed with 50 wt% of amphibolite chips (BA5) in a graphite crucible, heated at 1200 °C for 15 min, and slow cooled at 10 °C min^−1^. To test the effects of mixed granite and amphibolite, 50 wt% each of chips of amphibolite (BA3) and granitoid (#443, similar composition to RG, see Table S3) were mixed in a Pt crucible, heated at 1200 °C for 15 min, and cooled at 10 °C min^−1^.

A summary of all the experimental conditions is shown in Table 2.

### Characterization

#### Chemistry of source rocks

Whole-rock chemistry measurements for amphibolites and dolerite were previously obtained^57^. Compositions of various specific Columbia River Basalts (CRB) were obtained from records at the Washington State University Geoanalytical Laboratory. Select compositions are provided in Table 3, with all studied compositions listed in Table S3. Comparison of the studied volcanic^61^ and plutonic^62^ compositions is shown in the classical total alkali-silica (TAS) diagram for volcanics in the main text (Fig. 9).

**Table 3:** Major element compositions of selected source rocks for melting experiments (wt%).

	Amphibolite	Amphibolite	Dolerite/diabase	Basaltic trachyandesite	Basaltic trachyandesite	granite-gneiss	granite-gneiss
	Broborg	Broborg	Broborg	Pacific NW USA	Pacific NW USA	Broborg	Broborg
#	BA3 (site3)	BA5 (site5)	Dike	UMAT-1	HAS37	‘red granitoid’ (RG)	‘white granitoid’ (WG)
SiO_2_	52.80	46.00	50.91	54.35	53.69	67.84	68.69
Al_2_O_3_	16.25	18.45	11.75	13.67	13.48	14.99	15.71
FeO_t_	10.29	10.29	10.95	12.72	12.95	3.36	2.98
CaO	9.50	10.30	8.33	6.48	6.57	2.48	2.95
MgO	5.42	4.79	8.76	2.91	3.18	1.04	0.89
Na_2_O	2.17	1.75	2.74	3.29	3.44	3.76	3.59
K_2_O	1.27	1.67	1.02	2.64	2.64	3.92	3.66
TiO_2_	0.99	1.47	0.67	2.83	3.02	0.439	0.419
MnO	0.22	0.18	0.31	0.21	0.20	0.078	0.073
P_2_O_5_	0.21	0.46	0.18	0.90	0.82	0.128	0.124
Total	99.12	95.36	95.62	100.00	99.99	98.03	99.09
Technique	ICP-OES	ICP-OES	ICP-OES	XRF	XRF	XRF	XRF
Lab	SWRI	SWRI	SWRI	WSU	WSU	WSU	WSU

Note that FeO_t_ is FeO total, and redox conditions are not assumed. Additional rock compositions in Table S3. Chemical analysis was performed with X-ray fluorescence (XRF) or Inductively Coupled Plasma-Optical Emission Spectroscopy (ICP-OES).

#### Thermal analysis of source rocks

Differential thermal analysis (DTA) and thermogravimetric analysis (TGA) data were collected on selected samples using a SDT Q600–TA Instruments system in the temperature range of ~ 30 °C to 1300 °C and a heating rate of 10 °C min^−1^ under 100 mL min^−1^ air flow. Powdered samples of ~ 25 mg were tested in in alumina crucibles. Selected experiments were conducted measuring on heating then on subsequent cooling at 10 °C min^−1^ under the same air flow.

#### Microscopy

Petrographic analyses of archaeological samples and source rocks were performed on thin sections using standard polarizing light microscopes equipped with integrated digital cameras (see Section S1 and S2 for details). Selected thin sections were also observed by electron probe microanalysis (EPMA) at WSU using JEOL JXA-8500F microprobe paired with a Thermo UltraDry energy-dispersive spectroscopy (EDS) and five wavelength-dispersive spectrometers (WDS). The WDS measurements details are provided in the Table S1.

Selected unheated and heat-treated source rocks were investigated by scanning electron microscopy (SEM) with a back-scattered electron (BSE) detector to provide atomic number contrast and energy dispersive spectroscopy (EDS) to provide elemental information, using both Hitachi TM3030 and Oxford Instruments Quantax 70 EDX detectors. Samples were sectioned and mounted in epoxy resin, which was ground with subsequent 50 μm, 16 μm, 5 μm SiC grit paper and polished with 3 μm and 1 μm diamond paste suspensions, prior to being carbon coated.

Additionally, a more detailed microanalytical investigation was performed for BA3-1200-P-L-Q and BA5-1200-P-L-Q at 15 kV using a Hitachi S3700N SEM and an XFlash 6|60 Bruker silicon drift X-ray detector (see Section S6). Variable Z allowed the X-ray detector to be positioned to enable larger scanning areas and take-off angles. Hyperspectral X-ray images were deconvoluted for overlapping peaks and a polygon tool used to extract spectra for each chemical phase present. Ten glass compositions for each experiment were processed using a peak-background ZAF standardless matrix correction routine.

#### X-ray diffraction (XRD) and Rietveld refinement

##### Ex situ XRD

XRD of ex situ archaeological samples, source rocks, and experimentally melted rocks was performed using an X-ray diffractometer outfitted with a Co Kα X-ray tube. Rietveld refinement was performed on powdered materials either without an internal standard, but with a predetermined background, or with a corundum standard. Detailed methodology and data are contained in Section S3.

##### In situ hot stage XRD of source rocks

In situ hot-stage XRD of powder samples was carried out using an XRD outfitted with a Cu Kα X-ray tube and a platinum heating stage apparatus. Samples were crushed and powder was deposited onto the heating strip using ethanol and a pipette. In situ diffraction measurements were performed on selected samples (BA5 amphibolite, dike dolerite, HAS37 basalt, RG red gneiss) both while heating from room temperature and while cooling from elevated temperature. In-situ heating was from room temperature to 1200 °C at 10 °C min^−1^ with 1 h dwells at 200 °C, 500 °C, 700 °C, 850 °C, 950 °C, 1050 °C, and 1200 °C to allow for XRD measurements. In situ cooling trials were carried out by heating samples from room temperature to 1500 °C at 10 °C s^−1^ then cooling from 1500 °C to room temperature at 10 °C min^−1^ with 1 h dwells at 1450 °C and 50 °C increments from 1250 °C-800°C to allow for XRD measurements. For details see Section S4.

Rietveld refinement profile fitting was performed, but the addition of an internal standard would likely interact and alter the chemistry throughout the duration of the high temperature measurement; therefore, another method was developed to estimate the amorphous fraction as a function of temperature. The method selected was based on the Johnson–Mehl–Avrami–Kolmogorov (JMAK) equation, using the peak area of a given crystalline phase—feldspar for amphibolite and dolerite, magnetite for basalt—normalized to the average. After fitting the JMAK equation, the resulting functions were used to compute a temperature-specific correction factor which was multiplied to all phases, and the amorphous fraction in the sample was found by difference. Mathematics and details of this correction are provided in the Section S4.1.

#### Simulation of mineralogy

To simulate the predicted equilibrium phase distribution of the rocks tested for their melting, the rhyolite-MELTS software package was used^38^. Because the primary conditions of interest were melting at atmospheric pressure, a 1 bar condition was used, and the input compositions were those of Table 2, thus neglecting any water which would escape at atmospheric pressures. For reduction–oxidation (redox) conditions, two buffers were chosen to bound the problem, the FMQ (fayalite–magnetite–quartz) and the more oxidizing Hm-Mt (hematite–magnetite). We expect that an ambient temperature and pressure melting would be closer to an Hm-Mt buffer, but it is possible that portions deep in the wall or near charcoal could experience more reducing conditions. Results of the simulations are shown in Section S5.

## Supplementary Information

Supplementary Information
