# Supplementary material for: Reproduction of melting behavior for vitrified hillforts based on amphibolite, granite, and basalt lithologies
Source: Sci Rep. 2021 Jan 14;11:1272. doi: 10.1038/s41598-020-80485-w (PMC7809384; doi:10.1038/s41598-020-80485-w)
Supplement: Supplementary file 1 — Supplementary Information [file 41598_2020_80485_MOESM1_ESM.pdf]

SUPPLEMENTARY INFORMATION

Reproduction of Melting Behavior for Vitrified Hillforts based on Amphibolite, Granite, and Basalt Lithologies

John S. McCloy,[1,2,3,4*] José Marcial,[1,4] Jack S. Clarke,[3] Mostafa Ahmadzadeh,[1,2] John A. Wolff,[5] Edward P. Vicenzi,[6] David L. Bollinger,[2] Erik Ogenhall,[7] Mia Englund,[7] Carolyn I. Pearce,[4] Rolf Sjöblom,[8] Albert A. Kruger[9]

[1]School of Mechanical and Materials Engineering, Washington State University, Pullman, WA, USA

[2]Materials Science and Engineering Program, Washington State University, Pullman, WA, USA

[3]Department of Materials Science and Engineering, University of Sheffield, UK

[4]Pacific Northwest National Laboratory, Richland, WA, USA

[5]School of the Environment, Washington State University, Pullman, WA, USA

[6]Museum Conservation Institute, Smithsonian Institution, Suitland, MD, USA

[7]The Archaeologists, National Historical Museums (SHM), Uppsala, Sweden

[8]Luleå University of Technology, Luleå, Sweden

[9]US Department of Energy, Richland, WA, USA

*corresponding author, john.mccloy@wsu.edu

This file includes:

- Supplementary Text
- Table S-1 to S-9
- Fig. S-1 to S-40
- References

SUPPLEMENTARY INFORMATION

S1 Broborg samples

S1.1 Electron microscopy and microanalysis of Broborg samples

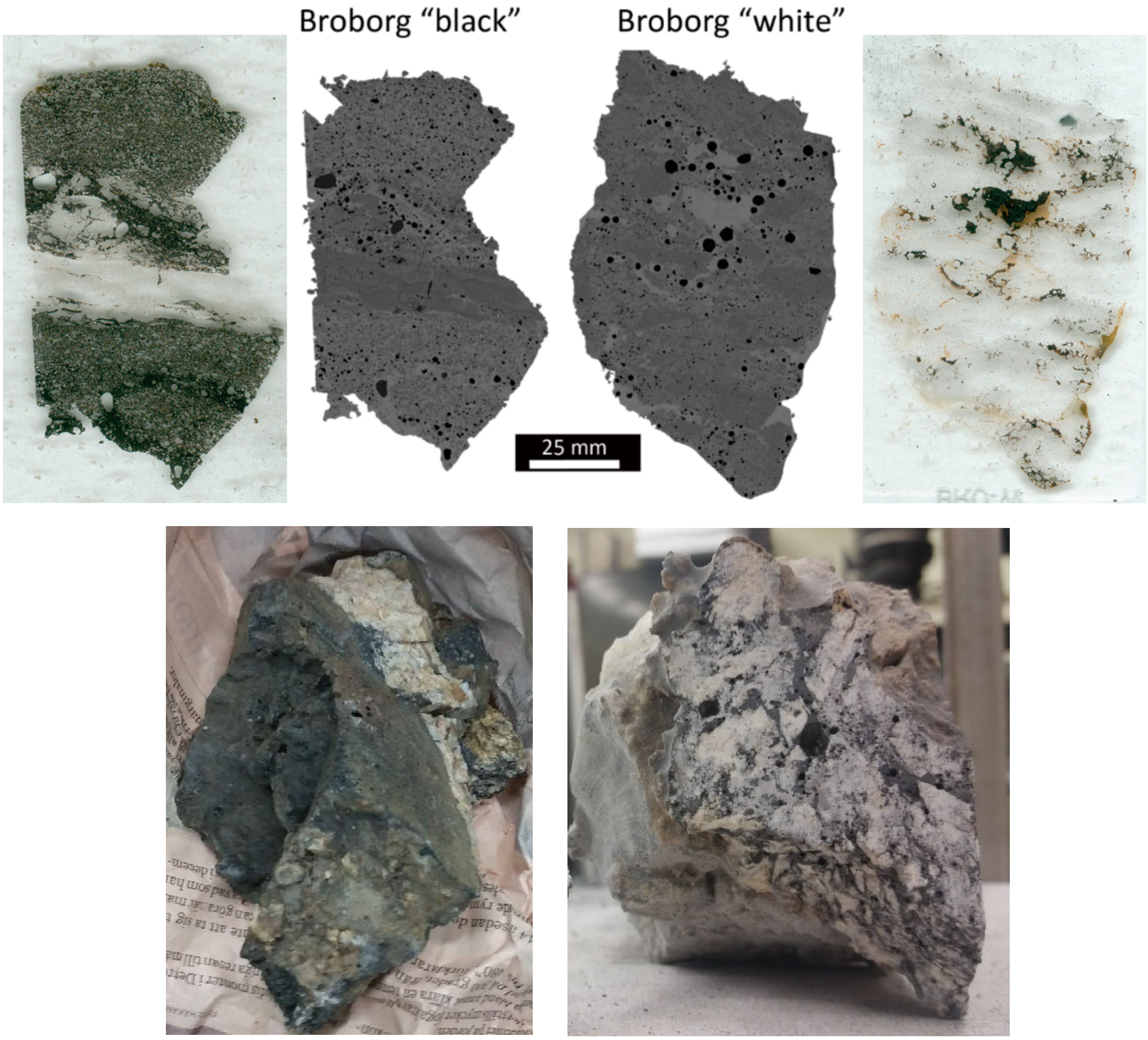

Fig S-1. Optical microscopy images (outer) and SEM (JEOL-COMPO) backscattered electron maps (inner) of Broborg black (Bro-B) and white (Bro-W) thin section samples; all thin section images obtained at WSU; below shows the rock samples from which these thin sections were obtained (photos C. Pearce).

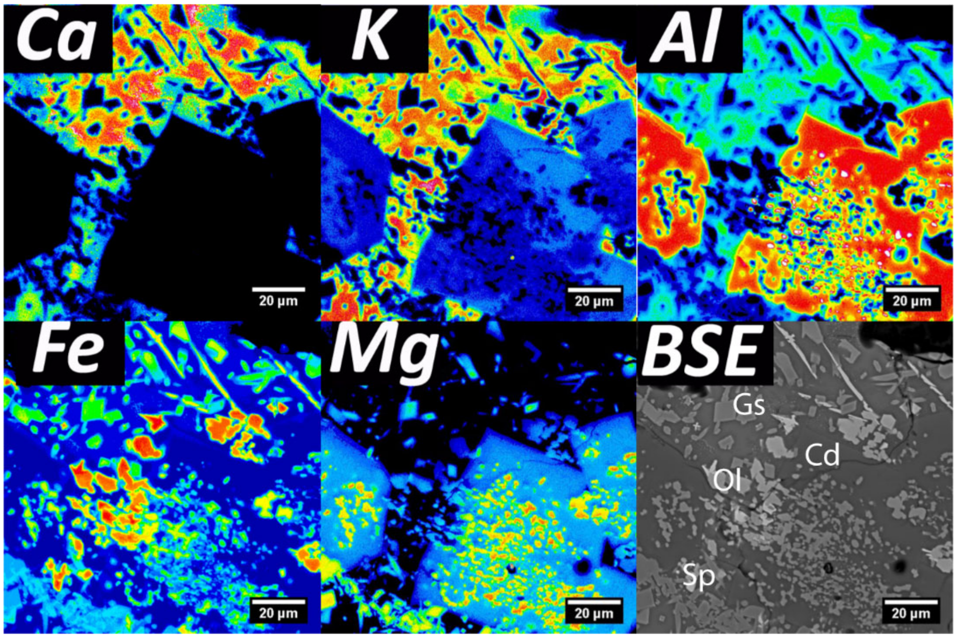

Fig S-2. WDS map (obtained at WSU) of Bro-B exhibiting cordierite (Cd), spinel (Sp), olivine (Ol), and glass (Gs) (Map #1). Red is high concentration and blue is low concentration.

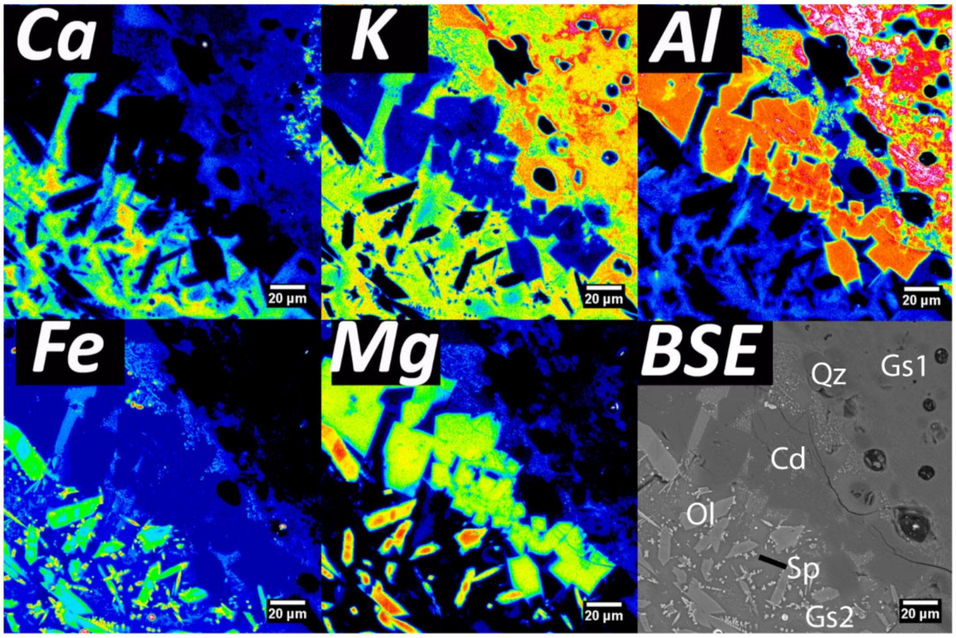

Fig. S-3. WDS map (obtained at WSU) of Bro-B exhibiting olivine (Ol), cordierite (Cd), spinel (Sp), and two different residual glasses (Gs1, Gs2) (Map #2). Red is high concentration and blue is low concentration.

S1.2 Electron probe microanalysis (EPMA) linescan of Bro-W

JEOL JXA-8500F, WSU GeoAnalytical Lab. ZAF (Armstrong-Love/Scott) intensity correction. 15 kV, 10 nA, 10 µm beam size.

Table S-1. EDS-WDS measurement conditions used for linescans

Oxide	Peak Counting Time (s)	Low Background Counting Time (s)	High Background Counting Time (s)	Spectrometer #	Analyzing Crystal	Calibration Standard
SiO_2	40*	EDS*	EDS*	EDS*	EDS*	K-412 NIST Glass
TiO_2	60	30	30	1	PETJ	Sphene #1A (C.M. Taylor)
Al_2O_3	40*	EDS*	EDS*	EDS*	EDS*	Anorthite, Great Sitkin (NMNH 137041)
FeO	20	10	10	3	LiFH	Fayalite, Rockport (NMNH 85276)
MnO	40	20	20	3	LiFH	Spessartine (C.M. Taylor)
MgO	20	10	10	5	TAP	K-411 NIST Glass
CaO	20	10	10	2	PETJ	K-411 NIST Glass
Na_2O	15	7.5	7.5	5	TAP	Albite #4 (C.M. Taylor)
K_2O	40	20	MAN	1	TAP	Orthoclase, OR-1 (Ingamells)
P_2O_5	60	30	30	4	PETJ	Apatite, Wilburforce (C.M. Taylor)

*Acquired using Thermo UltraDry EDS spectrometer. Peak counting time is dead-time corrected.

Table S-2. SEM-WDS/EDS line scans along two traces of the Broborg "white" sample; BDL=below detectability limit

distance (µm)	SiO_2	TiO_2	Al_2O_3	FeO	MnO	MgO	CaO	Na_2O	K_2O	P_2O_5	Total
**Trace #1**											
**0**	66.46	BDL	19.07	1.02	0.03	0.33	1.22	3.09	9.11	BDL	100.33
**130**	49.63	2.42	17.34	12.36	0.42	5.01	5.96	2.63	3.15	0.65	99.58
**Trace #2**											
**0**	66.86	BDL	19.67	1.33	0.04	0.41	1.10	3.67	7.51	BDL	100.60
**190**	54.88	1.97	17.10	10.74	0.34	4.41	4.36	3.06	2.82	0.36	100.03

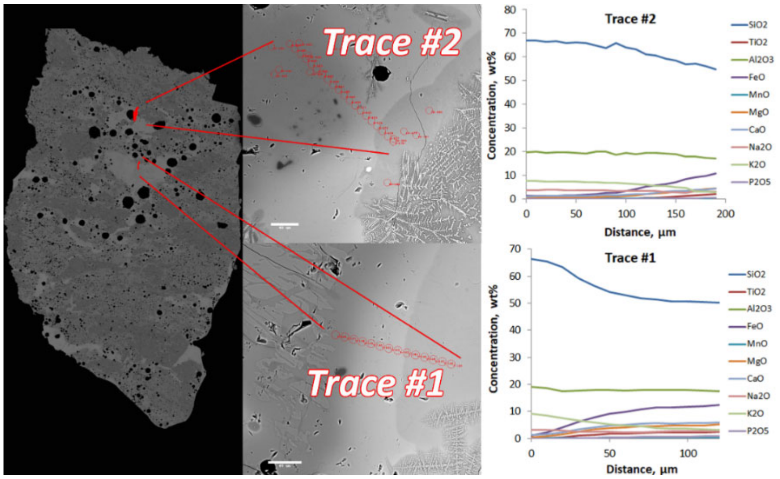

Fig S-4.WDS-EDS linescan in Bro-W showing two compositionally different residual glasses in intimate contact

S2 Source Rocks

S2.1 Whole rock chemistry

Table S-3. Whole rock chemistry of selected rocks (in wt.%)

	UMAT-1	HAS37	BGR (15.1.13)	DIKE	BA1	BA2	BA3	BA4	BA5	BB6	White gneiss1 (WG)	442	Red gneiss (RG)	443
	Basalt	Basalt	Basalt	Dolerite	Amphibolite	Amphibolite	Amphibolite	Amphibolite	Amphibolite	Amphibolite	Granitoid	Granitoid	Granitoid	Granitoid
SiO_2	54.35	53.69	55.79	50.91	53.20	61.80	52.80	53.60	46.00	45.50	68.69	69.83	67.84	69.42
Al_2O_3	13.67	13.48	14.15	11.75	17.15	16.65	16.25	17.40	18.45	15.80	15.71	15.19	14.99	15.03
FeO*	12.72	12.95	11.18	10.95	10.29	10.29	10.29	10.29	10.29	10.29	2.98	3.13	3.36	3.07
CaO	6.48	6.57	7.44	8.33	6.40	5.10	9.50	7.44	10.30	7.90	2.95	2.47	2.48	2.81
MgO	2.91	3.18	3.82	8.76	2.98	1.94	5.42	3.99	4.79	10.9	0.89	1.02	1.04	0.85
Na_2O	3.29	3.44	3.40	2.74	2.56	2.81	2.17	2.46	1.75	0.89	3.59	3.53	3.76	3.45
K_2O	2.64	2.64	1.68	1.02	2.32	2.24	1.27	1.91	1.67	1.61	3.66	3.54	3.92	3.62
TiO_2	2.83	3.02	2.06	0.67	1.45	0.82	0.99	1.13	1.47	0.63	0.419	0.430	0.439	0.399
MnO	0.21	0.20	0.18	0.31	0.20	0.09	0.22	0.17	0.18	0.22	0.073	0.075	0.078	0.070
P_2O_5	0.90	0.82	0.30	0.18	0.44	0.22	0.21	0.31	0.46	0.09	0.124	0.141	0.128	0.125
TOTAL	100.00	99.99	100.00	95.62	96.99	101.96	99.12	98.7	95.36	93.83	99.09	99.36	98.03	98.85
Typical grain size (mm)					0.1	0.2-0.3; 1 pc	≤0.1 - 1	≤0.1 - > 2	0.2 - 1	0.5 - 2				

BGR = Basalt Grande Ronde, Columbia River Basalt Group
For grain size, n.d. indicates not determined; pc is size of large phenocryst; amphibolite grain sizes from Ogenhall.[S1]

S2.2 Thin sections

Petrographic analyses of archaeological samples and source rocks were performed on thin sections using an Olympus BX53-P (archaeological samples, dike, and granites) or Zeiss Axioskop 40A (amphibolites) polarizing light microscopes equipped with integrated digital cameras for continuous documentation of the analyses. Thin sections were prepared by Spectrum Petrographics (Vancouver, WA, USA) or Axinit (Bratislava, Slovakia).

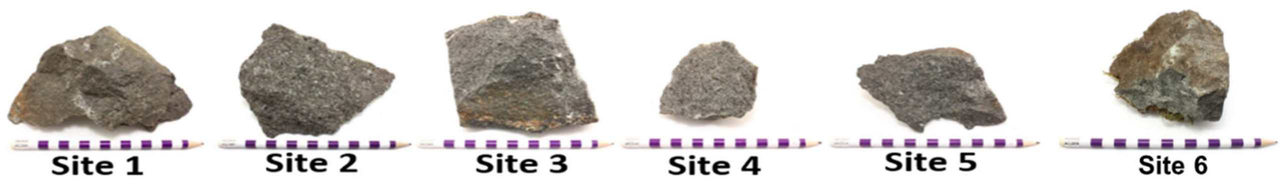

Fig. S-5. Images of hand samples of Broborg amphibolites. BA1-BA5 (i.e., site 1 – site 5) are from outcrops. BB6 (i.e., site 6) is from a boulder. See Ogenhall.[S1]

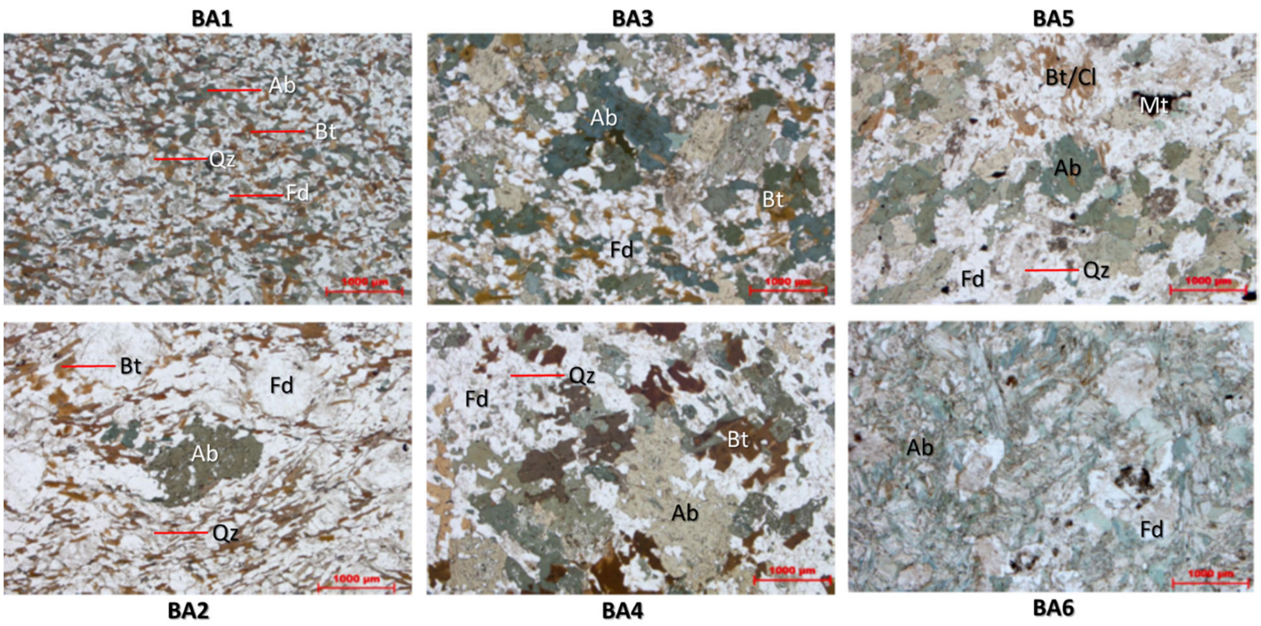

Fig. S-6. Transmitted plane polarized light photomicrographs of amphibolite samples: BA1 (site 1), BA2 (site 2), BA3 (site 3), BA4 (site 4), BA5 (site 5), BB6 (site 6). Scale bar is 1 mm. Labeled are minerals: quartz (Qz), amphibole (Ab), biotite (Bt), feldspar (Fd), magnetite (Mt)

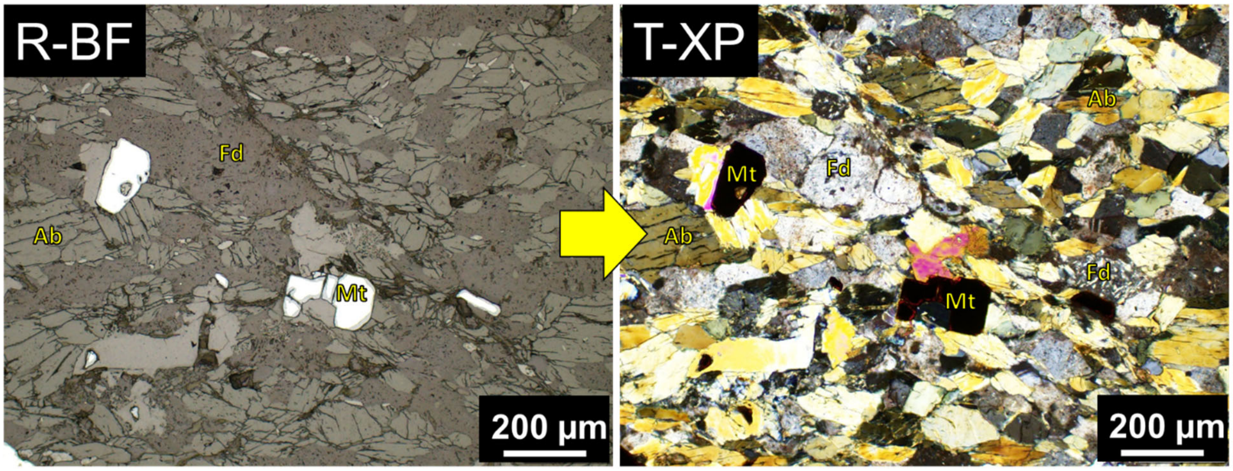

Fig. S-7. (R-BF) and transmitted light cross polarized (T-XP) photomicrographs of dolerite (dike) sample. Labeled are minerals: amphibole (Ab), feldspar (Fd), magnetite (Mt)

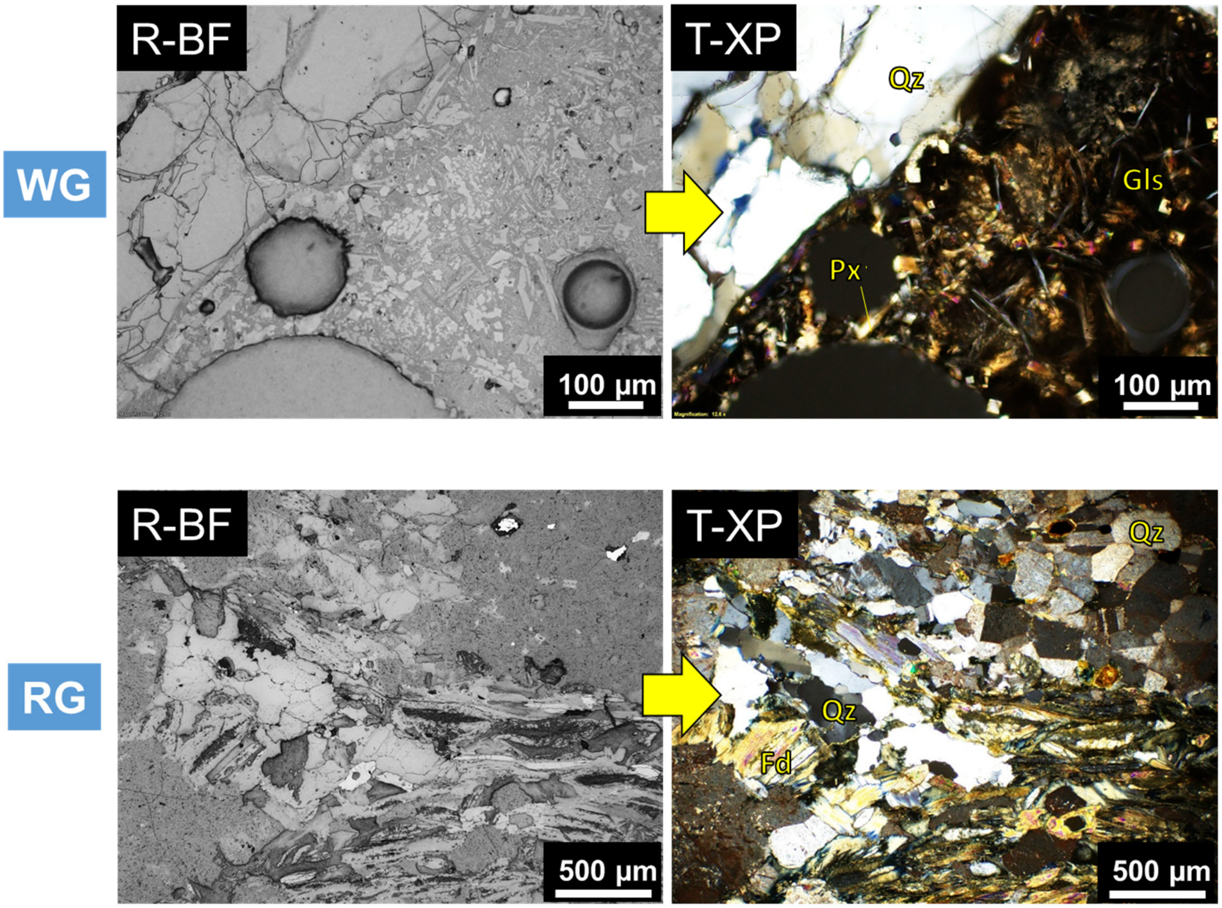

Fig. S-8. Optical microscopy; reflected light bright field (R-BF) and transmitted light cross polarized (T-XP) photomicrographs of white gneiss (WG) and red gneiss (RG) samples. Labeled are minerals: quartz (Qz), feldspar (Fd), pyroxene (Px), and glass (Gls). WG shows evidence of being previously heated and recrystallized. Granitoids "442" and "443" (see Table S-3) were also investigated. 442 was similar to WG and 443 was similar to RG.

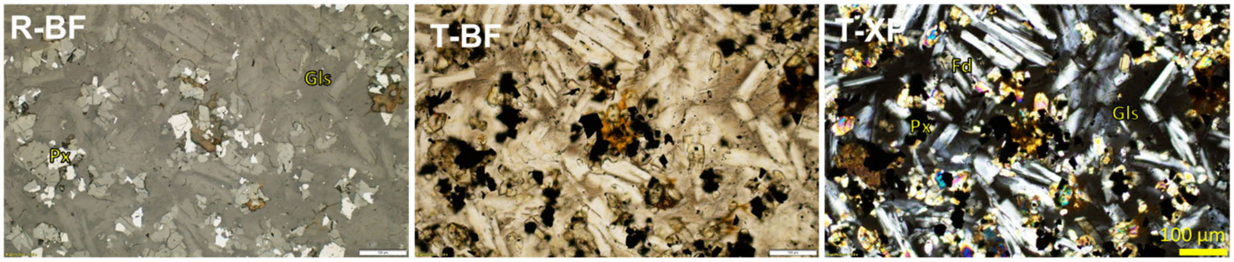

Fig. S-9. Optical microscopy; reflected light bright field (R-BF) and transmitted light cross polarized (T-XP) photomicrographs of basalt (HAS37) sample. Labeled are minerals: feldspar (Fd), pyroxene (Px), and glass (Gls). HAS37 is a very similar basalt to UMAT1 (see Table S-3).

S2.3 Thermal analysis

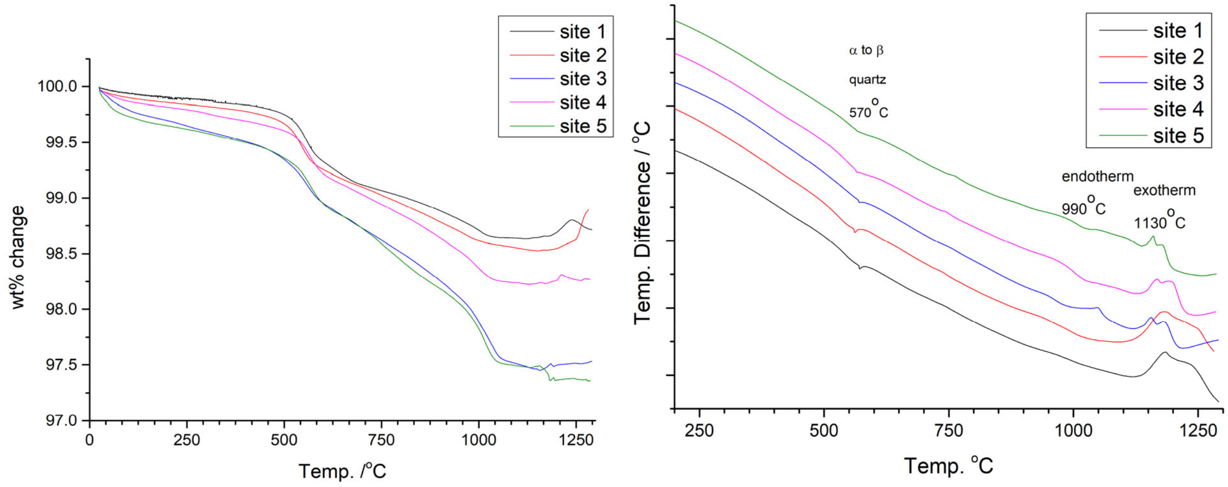

Fig. S-10. Thermal analysis scans on heating for amphibolite samples BA1-BA5 (i.e., site 1 – site 5). Left: thermogravimetric analysis (TGA); Right: Differential thermal analysis (DTA)

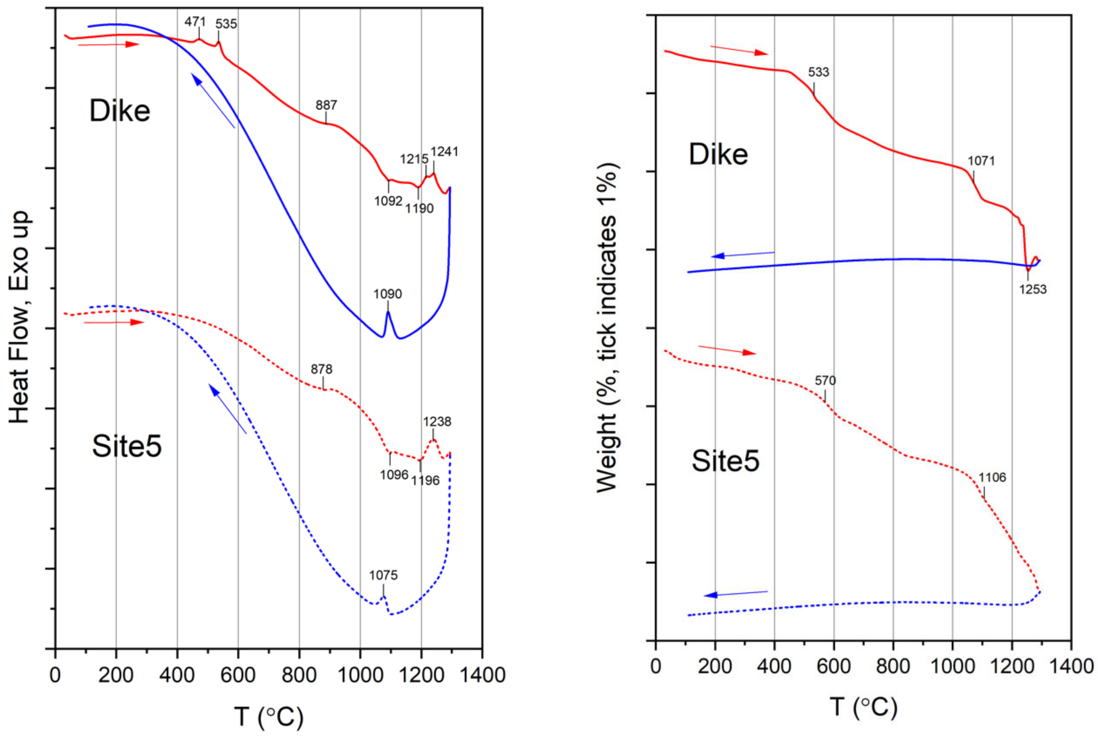

Fig. S-11. DTA and TGA results of dike (dolerite) and BA5 (site 5) source rocks on heating and cooling.

S3 *Ex situ* XRD and Rietveld refinement

S3.1 Methodology

Ex situ XRD. X-ray diffraction of *ex situ* archaeological samples, source rocks, and experimentally melted rocks was performed using a PANalytical MPD PRO X-ray diffractometer outfitted with a Co Kα X-ray tube with 40 kV accelerating voltage and 40 mA and an X'celerator detector. The scan parameters were 2-90° (Co) measurement range, 0.05° step size, 10 s dwell, and 15 repetitions per sample.

Rietveld refinement was performed using HighScore plus (PANalytical, Netherlands) on powdered materials either without an internal standard but with a predetermined background or with a 20 wt.% corundum standard. Refinements included scale factor, specimen displacement, unit cell, u/v/w parameters, peak shape, split width and shape, and preferred orientation (using spherical harmonics).

Some samples were also measured with a Cu Kα X-ray tube on the same instrument, and the resulting measurements and refinements were comparable.

Powders were obtained for source rocks by crushing to smaller sizes to obtain a uniform representation of the heterogeneous rocks. Note that this allows determination of average mineral composition of the rocks (this was particularly observed for BA3 which had large quartz seams). This should not be confused with melting experiments on powders; melting of large rocks of these large grain-size materials (> tens of micrometers) would necessarily be different than melting of powders.

S3.2 Amphibolites

The monolith samples were placed in Pt containers and placed into a furnace at 850°C or 1050°C, and left for 15 min before air quenching. Air quenching of monolithic samples, at 850 and 1050°C generally caused them to crumble, and this was determined to be due to thermal shock of the monoliths rather than from any reaction of the phases.

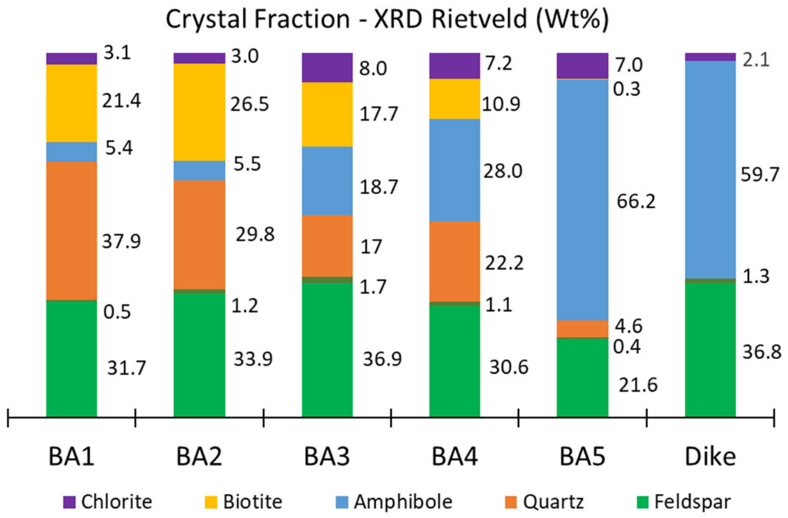

Fig. S-12. Rietveld quantitative XRD phase analysis (wt.%) of selected amphibolite and related starting rocks. All data from Co Kα. For fitting: feldspar (albite majority, microcline trace); biotite (phlogopite); amphibole (actinolite); chlorite (clinochlore); quartz (α quartz)

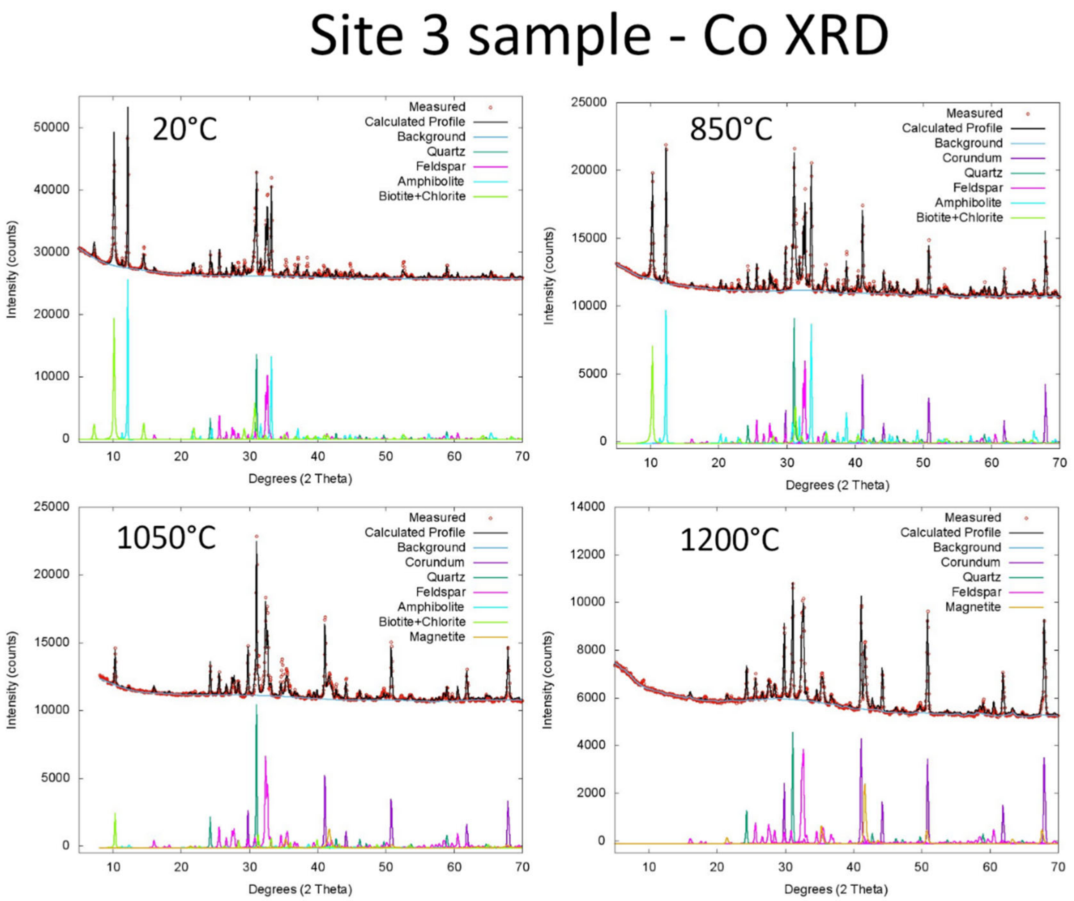

Fig. S-13. Rietveld refinements for BA3 (site 3) amphibolite with Co tube data. Room temperature assumed as 20°C. All samples except room temperature rock used an internal standard.

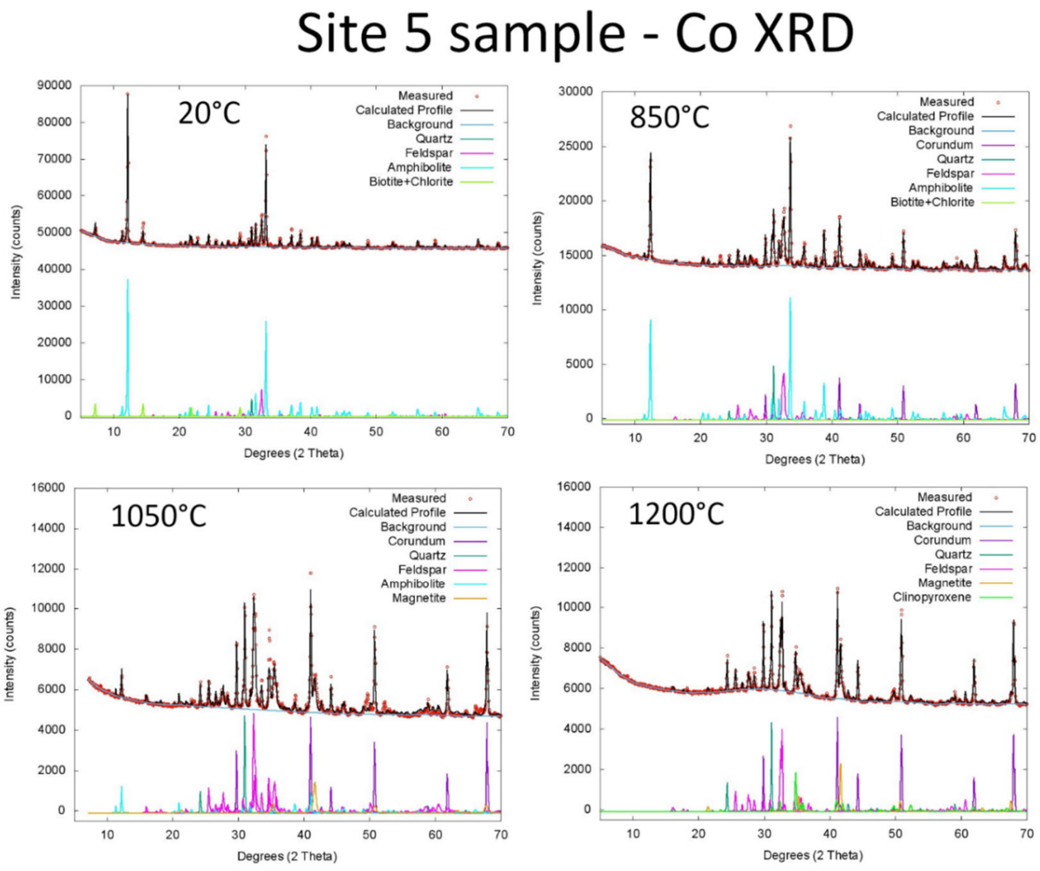

Fig. S-14. Rietveld refinements for BA5 (site 5) amphibolite with Co tube data. Room temperature assumed as 20°C. All samples except room temperature rock used an internal standard.

Table S-4. XRD analysis results (wt.%) for BA5 amphibolite at 1200°C with different crucible and cooling conditions. Notes (see Methods for details): 'slow' cooling is 10°C min^{-1}; 'chunks' are 2-3 cm monoliths; 'chips' are <3 mm particles.

**Name**	BA5-1200-P-L-SC-120	BA5-1200-P-L-SC	BA5-1200-P-S-SC	BA5-1200-P-S-Q	BA5-1200-G-S-Q	BA5-1200-G-S-SC
**Heat treat (min)**	120	15	15	15	15	15
**Crucible**	Pt	Pt	Pt	Pt	Graphite	Graphite
**Sample size**	Chunk	Chunk	Chips	Chips	Chips	Chips
**Cooling**	Slow	Slow	Slow	Quench	Quench	Slow
Amorphous	43.5	44.9	41.7	77.6	87.5	30.6
Quartz	1.0	0.7	3.2	1.5	1.1	1.2
Albite	19.1	19.1	19.1	14.9	10.3	37.6
Microcline	1.4	1.2	0.7	1.7	1.2	0.3
Magnetite	6.2	7.5	8.6	4.3	0.0	0.7
Hematite	0.0	0.6	0.0	0.0	0.0	0.0
Clinopyroxene	28.7	26.1	26.7	0.0	0.0	19.5
Olivine	0.0	0.0	0.0	0.0	0.0	10.2
Total	99.9	100.1	100.0	100.0	100.1	100.1

Table S-5. XRD data (Co tube) for amphibolites XX-YY-P-L-Q. Room temperature assumed as 20°C.

Sample number	Amphibolite rock	X-ray tube	Internal standard	Dwell T (°C)	Amorphous	Quartz	Amphibole (Actinolite)	Feldspar (Total)	Biotite (Phlogopite)	Clino-pyroxene (Augite)	Magnetite	Other
BA1-20-P-L-Q	BA1	Co	N	20	0	37.9	5.4	32.2	24.5	0	0	0
BA1-850-P-L-Q	BA1	Co	N	850	0	45.9	0	49.8	4.3	0	0	0
BA1-1050-P-L-Q	BA1	Co	Y	1050	23.7	15.7	0	43.5	10.3	0	6.8	0
BA1-1200-P-L-SC	BA1	Co	Y	1200	13.6	31.6	0	46.8	0	3.1	3.1	1.9 (hematite)
BA1-1200-P-L-SC	BA1	Cu	Y	1200	13.9	35.2	0	47.5	0	3.0	0.4	
BA2-20-P-L-Q	BA2	Co	N	20	0	29.8	5.5	35.1	29.5	0	0	0
BA2-850-P-L-Q	BA2	Co	Y	850	19.6	7.3	51.1	21.9	0	0	0	0
BA2-1050-P-L-Q	BA2	Co	Y	1050	21.3	27.8	0	39.5	6.8	0	4.6	0
BA2-1200-P-L-SC	BA2	Co	Y	1200	24.7	28.5	0	37.8	0	1.2	5.6	2.1 (hematite)
BA2-1200-P-L-SC	BA2	Cu	Y	1200	24.4	35.3	0	37.4	0	2.1	0.3	0
BA3-20-P-L-Q	BA3	Co	N	20	0	17	18.7	38.6	25.7	0	0	0
BA3-850-P-L-Q	BA3	Co	Y	850	11.1	13.5	31	27.9	16.5	0	0	0
BA3-1050-P-L-Q	BA3	Co	Y	1050	39.0	13.4	2.9	35.4	3.1	0	6.2	0
BA3-1200-P-L-Q	BA3	Co	Y	1200	63.3	7.7	0	21.6	0	0	7.3	0
BA3-1200-P-L-SC	BA3	Co	Y	1200	32.3	6.9	0	38.0	0	12.1	9.2	1.6 (hematite)
BA3-1200-P-L-SC	BA3	Cu	Y	1200	42.9	6.8	0	34.5	0	14.6	0.8	0
BA4-20-P-L-Q	BA4	Co	N	20	0	22.2	28.0	31.7	18.1	0	0	0
BA4-850-P-L-Q	BA4	Co	Y	850	11.3	16.8	24.6	35.9	11.4	0	0	0
BA4-1050-P-L-Q	BA4	Co	Y	1050	44.6	18.3	0.2	28.8	3.4	0	4.6	0
BA4-1200-P-L-SC	BA4	Co	Y	1200	36.1	8.2	0	36.6	0	9.9	6.2	3.1 (hematite)
BA4-1200-P-L-SC	BA4	Cu	Y	1200	39.0	6.5	0	37.6	0	15.9	1.0	0
BA5-20-P-L-Q	BA5	Co	N	20	0	4.6	66.2	22.0	7.3	0	0	0
BA5-850-P-L-Q	BA5	Co	Y	850	23.0	6.4	43.6	26.9	0.1	0	0	0
BA5-1050-P-L-Q	BA5	Co	Y	1050	39.9	6.2	5.8	41.6	0	0	6.5	0
BA5-1200-P-L-Q	BA5	Co	Y	1200	60.3	6.1	0	19.4	0	8.7	5.5	0
BA5-1200-P-L-SC	BA5	Co	Y	1200	44.9	0.7	0	20.3	0	26.1	7.5	0
BA5-1200-P-L-SC	BA5	Cu	Y	1200	43.9	2.6	0	23.7	0	28.4	1.5	0
BA5-1200-P-L-SC-120dw	BA5	Cu	Y	1200	41.9	0.8	0	22.6	0	33.2	1.5	0

Table S-6. XRD data for amphibolites: Graphite versus Platinum crucibles: Cu Kα vs Co Kα data.

Internal standard used to quantify amorphous fraction. On select samples, both Cu and Co spectra were obtained. Rietveld refinement was performed by two different people. Even with choice of different feldspar phases, the overall results were nearly identical within analytical error and fitting error.

Sample number	Amphibolite rock	X-ray tube	Internal standard	Dwell T (°C)	Amorphous	Quartz	Amphibole (Actinolite)	Feldspar (Total)	Biotite (Phlogopite)	Clino-pyroxene (Augite)	Magnetite	Other
BA5-1200-P-S-Q	BA5	Co	Y	1200	77.6	1.5	0	14.9 (albite) 1.7 (microcline)	0	0	4.3	0
BA5-1200-P-S-Q	BA5	Cu	Y	1200	80.5	1.5	0	15.2 (anorthite)	0	0.2	02.5	0
BA5-1200-P-S-SC	BA5	Co	Y	1200	41.7	3.2	0	19.1 (albite) 0.7 (microcline)	0	26.7	8.6	0
BA5-1200-P-S-SC	BA5	Cu	Y	1200	49.1	1.6	0	21.0 (anorthite)	0	26.7	1.5	0.0
BA5-1200-G-S-Q	BA5	Co	Y	1200	87.5	1.1	0	10.3 (albite) 1.2 (microcline)	0	0	0	0
BA5-1200-G-S-Q	BA5	Cu	Y	1200	86.7	2.0	0.0	11.4 (anorthite)	0	0	0	0
BA5-1200-G-S-SC	BA5	Co	Y	1200	30.6	1.2	0	37.6 (albite) 0.3 (microcline)	0	19.5	0.7	10.2 (olivine)
BA5-1200-G-S-SC	BA5	Cu	Y	1200	36.6	1.5	0	35.5 (anorthite)	0	17.0	0.6	8.7 (olivine)
BA3-1200-P-S-SC-gran	BA3	Co	N	1200	-	21.4	0	49.9	0	19.7	8.0	1.0 (hematite)
BA3-1200-P-S-SC-gran	BA3	Co	Y	1200	26.1	14.4	0	25.7	0	28.6	5.3	0
BA5-1200-G-S-SC-clay	BA5	Co	N	1200	-	56.3	0	43.7	0	0	0	0
Broborg clay	-	Co	N	20	-	40.8	0	49.9	9.4 (muscovite)	0	0	0

- Clinopyroxene phase (Cu refinement) is ~Augite PDF# 98-041-7173, $Ca_{0.977}Fe_{0.187}Mg_{0.834}Na_{0.023}Si_2O_6$ from [S2]
- Olivine phase (Cu refinement) is ~Forsterite PDF# 98-009-1813, $FeMgSiO_4$ from [S3]

S3.3 Dolerite

Table S-7. XRD Rietveld refinement data for dolerite.

**Sample number**	**Dike**	**X-ray tube**	**Internal standard**	**Dwell T (°C)**	**Amorphous**	**Amphibole**	**Clino-pyroxene** *	**Spinel (magnetite)**	**Hematite**	**Feldspar (total)**	**Other**
D-20-P-L-Q	Dike	Cu	N	20	0	54.7<br>(ferropargasite)				45.3<br>(albite)	
D-850-P-L-Q	Dike	Cu	N	850	0	55.7<br>(tschermakite)				44.3<br>(albite)	
D-1050-P-L-Q	Dike	Cu	N	1050	0		47.4			52.6<br>(albite)	
D-1200-P-L-Q	Dike	Cu	Y	1200	86.3		13.7			0	
						(actinolite)#					
D-20-P-L-Q	Dike	Co	N	20	0	69.5	0	0	0	21.4<br>(albite)<br>5.0<br>(microcline)	4.1<br>(clinochlore)
D-850-P-L-Q	Dike	Co	N	850	0	72.3	0	0	0	23.5<br>(albite)<br>4.2<br>(microcline)	
D-850-P-L-Q	Dike	Co	Y	850	14.0	56.9	0	0	0	28.7<br>(albite)<br>0.5<br>(microcline)	
D-1050-P-L-Q	Dike	Co	Y	1050	45.0	0	21.8	4.2	0	21.5<br>(albite)<br>7.5<br>(microcline)	
D-1200-P-L-Q	Dike	Co	Y	1200	85.1	0	9.3	0	3.4	2.2 (albite)	

*fit with 98-000-9672: Diopside ($CaMgSi_2O_6$); Papike, J.J.;Appleman, D.E.;Clark, J.R., *American Mineralogist*, 58, 594 - 618, (1973)

#fit with 98-006-8319: Actinolite ($H_2Al_{0.23}Ca_{1.68}Fe_{0.48}Mg_{4.59}Na_{0.1}O_{24}Si_{7.92}$); Polosin, A.V.;Yamnova, N.A.;Zver'kova, O.N. (Zverkova, O.N.);Urusov, V.S., Vestnik Moskovskogo Universiteta, *Geologiy*a, 42, 43 - 53, (1987)

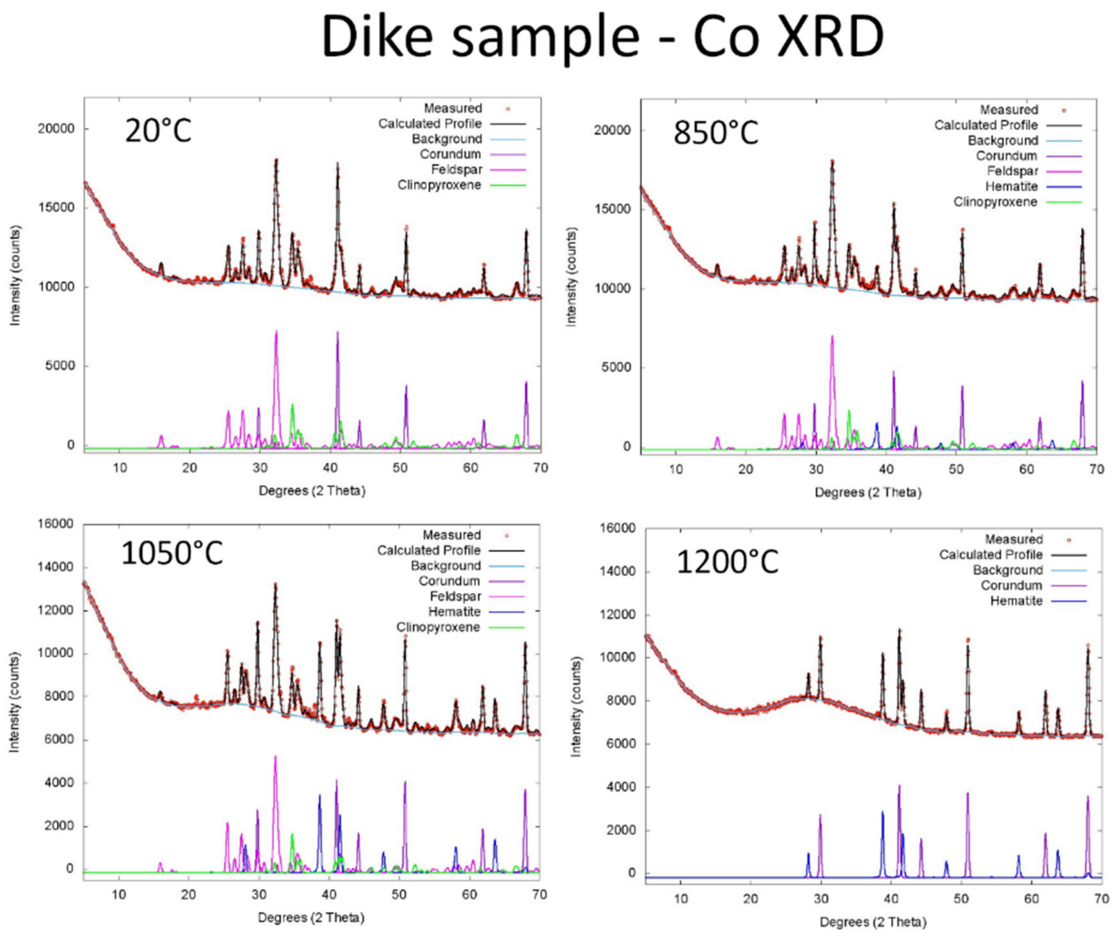

Fig. S-15. Rietveld refinements for dolerite (dike) with Co tube data. Room temperature assumed as 20°C. All samples except room temperature rock used internal standard.

S3.4 Basalts

Starting material for UMAT1 was powdered rock while for BGR it was chips. All were put in at temperature, held for 15 minutes, then removed from the furnace to air quench. Room temperature is assumed to be 20°C.

Table S-8. XRD Rietveld refinement data for basalt

Sample number	Basalt rock	X-ray tube	Internal standard	Dwell T (°C)	Feldspar (total)	Clino-pyroxene (Augite)	Hematite	Amorphous
U-20-P-S-Q	UMAT1	Co	Y	20	44.3 (albite+microcline)	18.6	0	37.1
U-850-P-S-Q	UMAT1	Co	Y	850	42.4 (albite+microcline)	15.3	6.7	35.7
U-1050-P-S-Q	UMAT1	Co	Y	1050	31.3 (albite+microcline)	10.6	10.8	47.3
U-1200-P-S-Q	UMAT1	Co	Y	1200	0	0	8.1	91.9
					**Feldspar (Anorthite)**			
U-20-P-S-Q	UMAT1	Cu	N	20	69.5	30.5	0	-
U-850-P-S-Q	UMAT1	Cu	N	850	70.5	21.4	8.1	-
U-1050-P-S-Q	UMAT1	Cu	N	1050	62.6	18.5	18.9	-
U-1200-P-S-Q	UMAT1	Cu	N	1200	0	0	100	-
U-1200-P-S-Q	UMAT1	Cu	Y	1200	0	0	6.7	93.3
BGR-20-P-S-Q	BGR 15.1.13	Cu	N	20	75.3	24.7	0	-
BGR-850-P-S-Q	BGR 15.1.13	Cu	N	850	73.0	27.0	0	-
BGR-1050-P-S-Q	BGR 15.1.13	Cu	N	1050	71.1	28.9	0	-
BGR-1200-P-S-Q	BGR 15.1.13	Cu	Y	1200	0	0	2.7	97.1

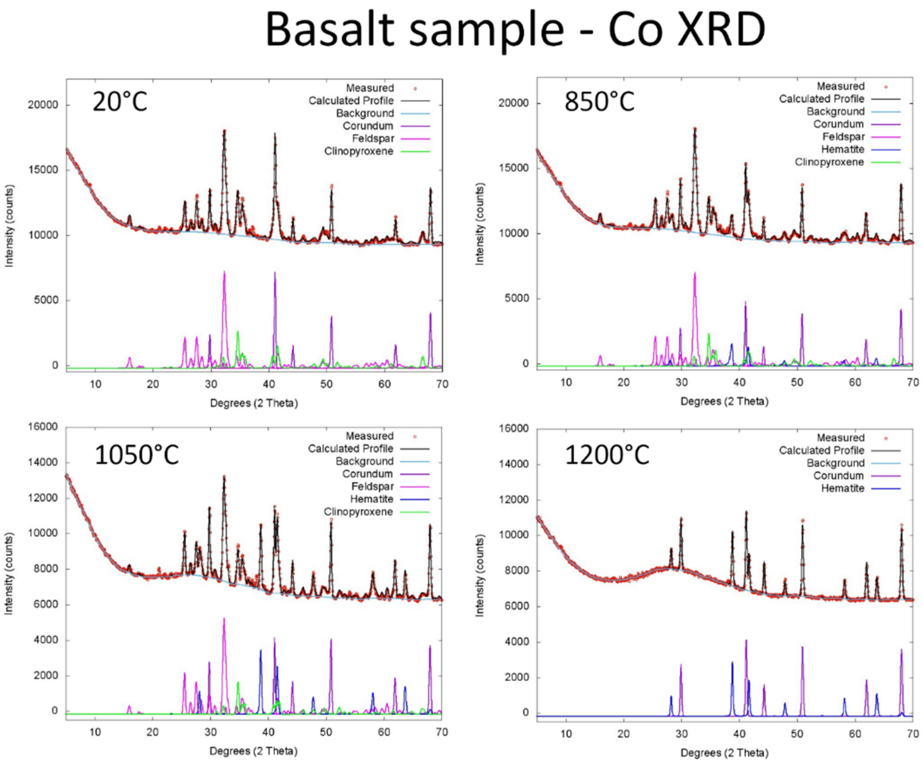

Fig. S-16. Rietveld refinements for basalt (UMAT1) with Co tube data. Room temperature is assumed as 20°C. All samples used an internal standard.

S3.5 Granitoids

Table S-9. X-ray diffraction Rietveld refinement data for granitoids

	**Granitoid rocks**	**X-ray tube**	**Internal standard**	**Dwell T (°C)**	**Amorphous**	**Quartz**	**Feldspar (total)**	**Feldspar (Albite)**	**Feldspar – Plagioclase**	**Feldspar – K-spar**	**Other**
									**(Bytownite)**	**(Microcline)**	
RG-20-P-L-Q	RG	Cu	N	20	-	32.9	67.0	47.5		19.5	
RG-850-P-L-Q	RG	Cu	N	850	-	35.2	64.8	46.9		17.9	
RG-1050-P-L-Q	RG	Cu	N	1050	-	38.3	61.7	44.6		17.1	
RG-1200-P-L-Q	RG	Cu	Y	1200	44.0	37.0	19.0		19.0	0	
									**(Anorthite)**	**(Anorthoclase)**	
WG-20-P-L-Q	WG	Cu	N	20	-	35.8	64.2		64.2		
WG-850-P-L-Q	WG	Cu	N	850	-	32.1	67.9			67.9	
WG-1050-P-L-Q	WG	Cu	N	1050	-	36.0	64.6		64.6		
WG-1200-P-L-Q	WG	Cu	Y	1200	23.9	36.2	39.9	39.9			
										**(Microcline)**	
RG-20-P-L-Q	RG	Co	N	20	-	24.8	72.6	47.1		25.5	2.6 (clinochlore)
RG-850-P-L-Q	RG	Co	N	850	-	26.4	73.6	50.7		22.9	
RG-1050-P-L-Q	RG	Co	N	1050	-	34.4	65.5	46.0		19.5	
RG-1050-P-L-Q	RG	Co	Y	1050	1.6	33.6	64.8	50.8		14.0	
RG-1200-P-L-Q	RG	Co	N	1200	-	52.1	47.9	29.1		18.8	
RG-1200-P-L-Q	RG	Co	Y	1200	50.2	29.4	20.4	13.4		7.0	
									**(Anorthite)**	**(Microcline)**	
WG-20-P-L-Q	WG	Co	N	20	-	28.0	72.0		51.3	20.7	
WG-850-P-L-Q	WG	Co	N	850	-	23.9	76.1		46.6	29.5	
WG-1050-P-L-Q	WG	Co	N	1050	-	26.7	73.3		51.5	21.8	
WG-1050-P-L-Q	WG	Co	Y	1050	30.2	18.5	51.3		28.3	23.0	
WG-1200-P-L-Q	WG	Co	N	1200	-	45.5	54.5		46.0	8.5	
WG-1200-P-L-Q	WG	Co	Y	1200	58.9	18.5	22.6		18.5	4.1	

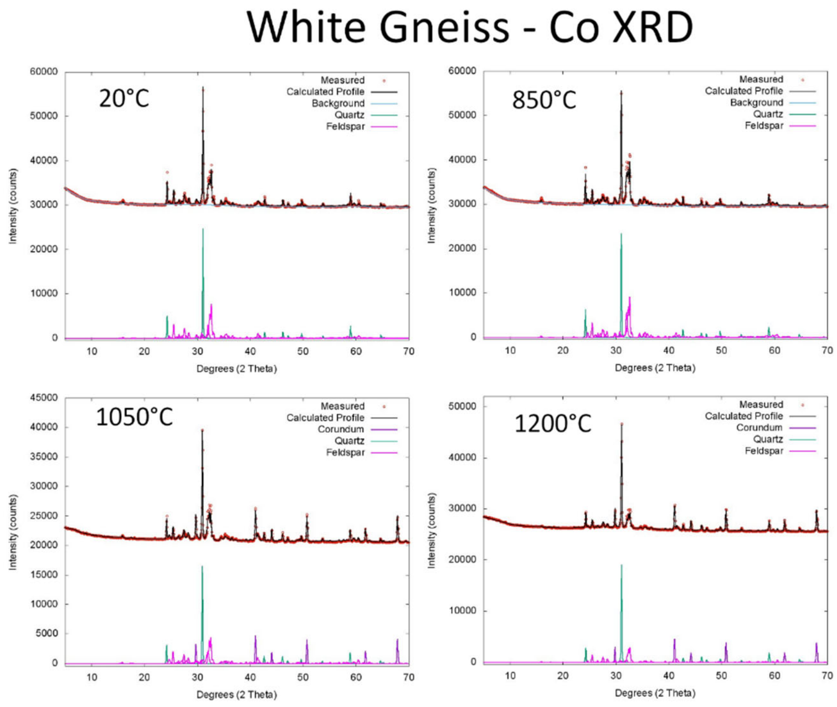

Fig. S-17. Rietveld refinements for granitoid (white gneiss) with Co tube data. Room temperature is assumed as 20°C. 1050°C and 1200°C samples used internal standard.

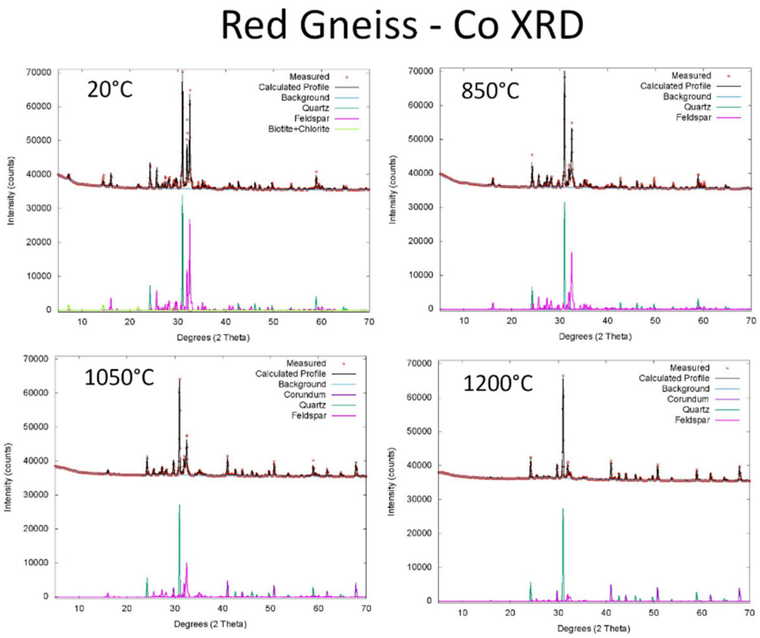

Fig. S-18. Rietveld refinements for granitoid (red gneiss) with Co tube data. Room temperature is assumed as 20°C. 1050°C and 1200°C samples used internal standard.

S4 In situ XRD

S4.1 Methodology

In situ hot stage XRD of source rocks. *In situ* hot-stage XRD of powdered samples was carried out using a Bruker D8 Advance X-ray diffractometer (XRD) outfitted with a Cu Kα X-ray tube (tube parameters: 45 kV and 40 mA) and a platinum heating stage apparatus. The scan parameters were 2-70° 2θ measurement range, 0.01° step size, 20 s dwell. Variable divergence slits were used to restrict the beam width to 10 mm, due to uneven heating beyond the center of the platinum heating strip caused by pre-existing thermomechanical creep, which led to necking of the heating strip (Fig. S-19). Samples were first crushed using an agate mortar and pestle then the powder was deposited onto the heating strip using ethanol and a plastic pipette.

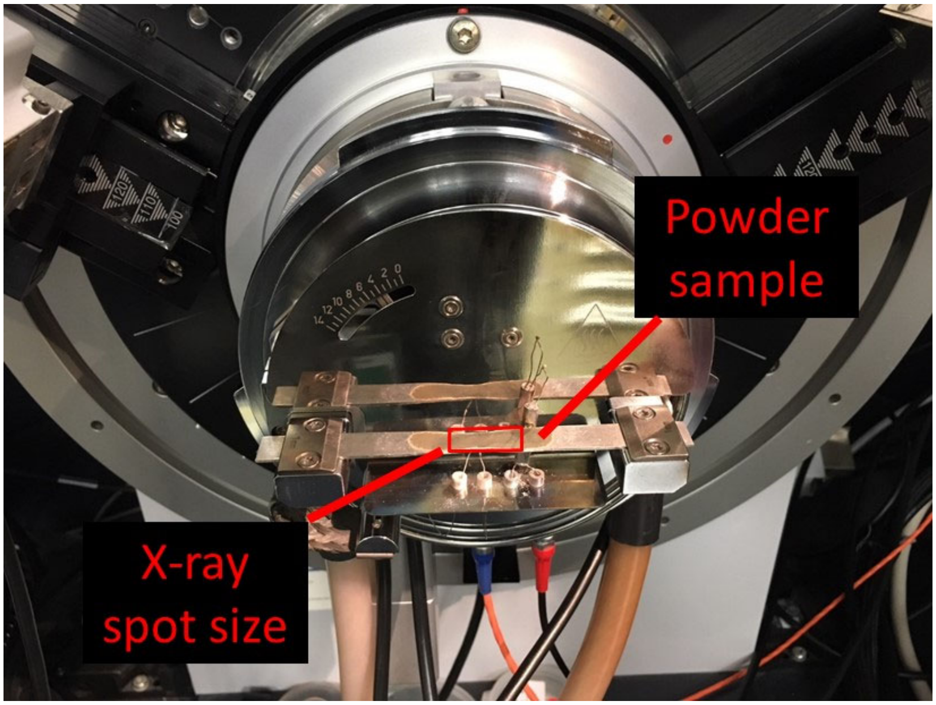

Fig. S-19. *In situ* XRD setup (photo J. Marcial)

In situ diffraction measurements were performed on selected samples (BA5 amphibolite, dike dolerite, HAS37 basalt) both while heating from room temperature and while cooling from elevated temperature. *In situ* heating trials were carried out by heating samples from room temperature to 1200°C at a heating rate of 10°C min^{-1} with 1 h dwells at 200, 500, 700, 850, 950, 1050, and 1200°C to allow for XRD measurements. *In situ* cooling trials were carried out by heating samples from room temperature to 1500°C at a heating rate of 10°C s^{-1} then cooling from 1500°C to room temperature at 10 C min^{-1} with 1 h dwells at 1450°C and 50°C increments from 1250-800°C to allow for XRD measurements. Fig. S-20 shows the cooling and heating profile used for this study.

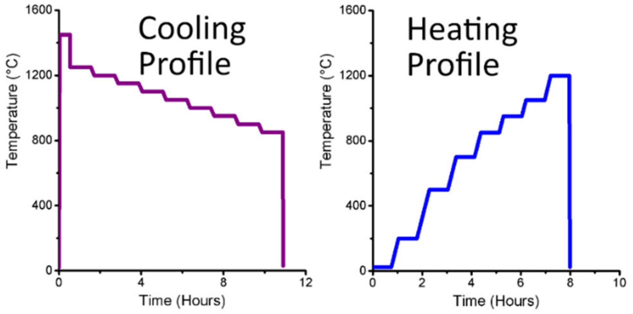

Fig. S-20. Cooling and heating profile for hot-stage XRD

Rietveld refinement and peak profile fitting was carried out using HighScore Plus (PANalytical) software. Any addition of an internal standard would likely interact and alter the chemistry throughout the duration of the high temperature measurement; therefore, another method had to be developed to estimate the amorphous fraction as a function of temperature (see Results). The method chosen first involved obtaining the peak area of one phase (anorthite) as a function of temperature which was then fit to the Johnson-Mehl-Avrami-Kolmogorov (JMAK) equation:

$$A/A_0 = e^{-(T/\tau)^n}$$ **Equation (1)**

where A/A_0 is the peak area of the anorthite phase normalized to the average—measured using a Calgliotti profile fitting function)—T is the dependent-variable temperature in degrees Kelvin), τ is the temperature at the inflection point (in degrees Kelvin), and n is the so-called Avrami exponent. The value of n was 17 for all samples and the τ value was 1480 K for BA5 amphibolite, 1368 K for the dike dolerite sample, and 1391 K for the HAS37 basalt. After fitting the JMAK equation, the resulting functions were used to compute a temperature-specific correction factor, F_T, such that $F_T=C_0(A/A_0)/C_T$, where C_0 is the average fraction of anorthite prior to melting and C_T is the fraction of anorthite at the specified temperature (i.e., 1050°C or 1200°C). The correction factor was multiplied to all phases and the amorphous fraction in the sample was found by the difference method.

This correction was modified for the case of the dike sample at 1200°C, where no anorthite was detected and the only phase present was magnetite. As a result, applying the correction factor would result in infinite magnetite. Therefore, the correction factor was instead taken as the value of A/A_0 from the JMAK fit for the temperature of 1200°C, which provided a reasonable estimate of the amorphous fraction at 1200°C for the dike sample.

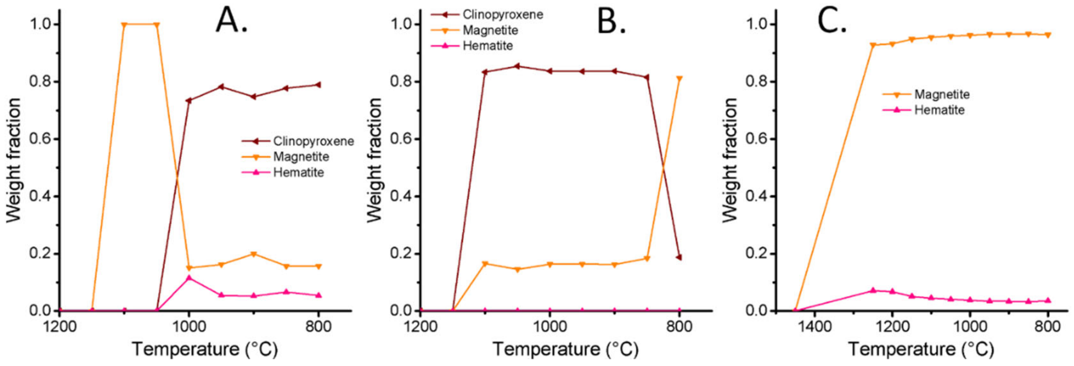

Fig. S-21. *In situ* hot-stage XRD experiments on cooling 1450°C to RT: A) amphibolite (BA5), B) dolerite (dike) C) basalt (HAS37).

S4.2 *In situ* vs. *ex situ* data, extended discussion

S4.2.1 Amphibolite (BA5)

Amphibole (hornblende) and feldspar have similar trends in both measurements. The amphibole fraction shows a sharp decrease at 950-1050°C (*in situ*), comparable to the large drop at 850-1050°C (*ex situ*), with phase fraction at zero at 1200°C in both measurements. Similarly, feldspar fraction drops at 1000°C in *in situ* but increases 1050°C and decreases 1200°C in *ex situ*. This difference is due to the requirement of fitting the feldspar to obtain the glassy (melt) fraction in the *in situ* run, as well as the fact that new feldspar forms on cooling in the *ex situ* experiment. To assess the minimum temperature for glass formation in the *ex situ* experiment, a 750°C data point was collected, showing no glass formation; thus melting begins between 750°C and 850°C. For the *in situ* experiment, better resolution can be seen regarding the breakdown of phyllosilicates (biotite, chlorite), which is absent between 500°C and 700°C—compare to Fig. 4 where some biotite remains after 15 min at 850°C. Also, quartz undergoes its low to high transition between 500°C and 700°C, which is not seen in the *ex situ* data since the quartz observed is always at room temperature (i.e., low quartz). In the *ex situ* experiment, some quartz still persists even after treatment at the highest temperatures, which is either related to kinetics (longer dwell times for *in situ*) or, less likely, due to new quartz being formed upon cooling.

A small amount of spinel (magnetite) is seen at the highest temperatures in the *in situ* experiment, with much more seen with *ex situ*, which is consistent with the finding above that most magnetite forms upon cooling. Similarly, no pyroxene is observed *in situ*, but is observed *ex situ*, since it forms on cooling. This result was again confirmed from the *in situ* XRD cooling measurements, where magnetite formed first upon cooling to 1100°C, with pyroxene and hematite forming at 1000°C. In the MELTS simulations (Fig S-22), which are equilibrium calculations, magnetite forms below 1400°C at the hematite-magnetite (Hm-Mt) buffer, but only below 1200°C for the fayalite-magnetite-quartz (FMQ) buffer. Hematite forms below 1100°C in Hm-Mt buffer, but only in trace amounts in FMQ buffer below 900°C. Pyroxene forms below 1200°C in both redox conditions, but in larger amounts in FMQ. Olivine only forms in FMQ conditions, below 1200°C, confirming the need for reducing conditions for olivine, as found in the *ex situ* experiments.

S4.2.2 Dolerite (dike)

In the case of the dolerite, the match between the amphibole and feldspar temperature trends is more similar for the two measurements. The formation of the melt is sharp between 950°C and 1050°C *in situ*, while some glass forms even at 850°C in the *ex situ* experiment, again similar to the amphibolite results; a 750°C data point showed no glass formation, thus melting begins between 750°C and 850°C. In the *in situ* results, the minor clinochlore phase is gone by about 550°C, and the minor quartz above 1050°C. High temperature phases include very minor spinel in the *in situ* case at 1200°C, whereas all feldspar and amphibolite have been destroyed. This was similar in the *ex situ* case, but spinel forms for the 1050°C max heat—likely upon cooling—but at 1200°C max heat the preferred iron oxide phase is hematite. Pyroxene only forms in the *ex situ* case, on cooling, with more forming when maximum temperature is 1050°C than when it is 1200°C.

In the *in situ* cooling experiments, both magnetite and pyroxene formed below 1100°C, with pyroxene being the more dominant phase—as determined by signal intensity—until the temperature dropped to 800°C, when magnetite became the dominant phase. In the MELTS simulations (Fig S-23), magnetite was the liquidus phase below 1400°C for Hm-Mt buffer, but olivine was the liquidus phase below 1350°C for FMQ buffer. Hematite was only predicted to form in the Hm-Mt buffer, below 1000°C. Thus, apparently the experiment was slightly oxidizing for the *ex situ* samples than for the *in situ* ones. In both redox conditions, feldspar and pyroxene crystallize below 1200°C. It should be noted that in no case was feldspar observed in the *in situ* cooling experiments of melted rocks, despite its prediction from equilibrium thermodynamics. This is due to the well-known problem of the difficulty of homogeneous nucleation of feldspar.[S4]

S5 MELTS simulations

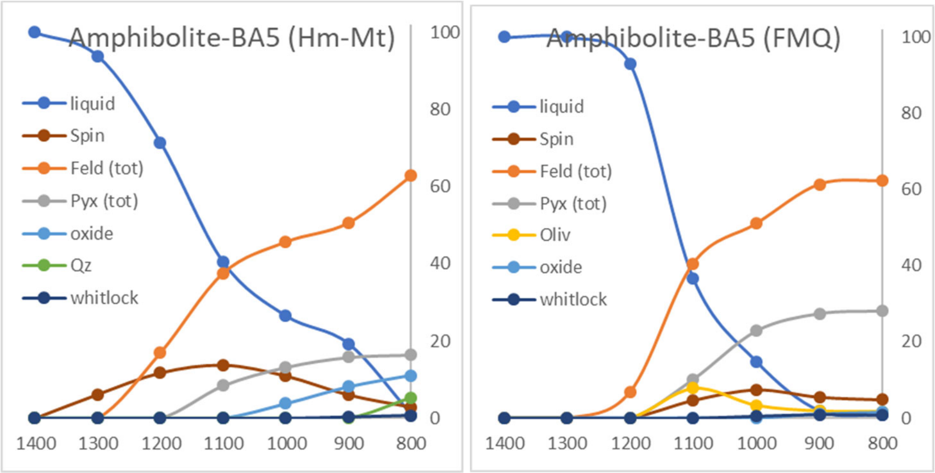

Fig. S-22. MELTS simulations for amphibolite (BA5)

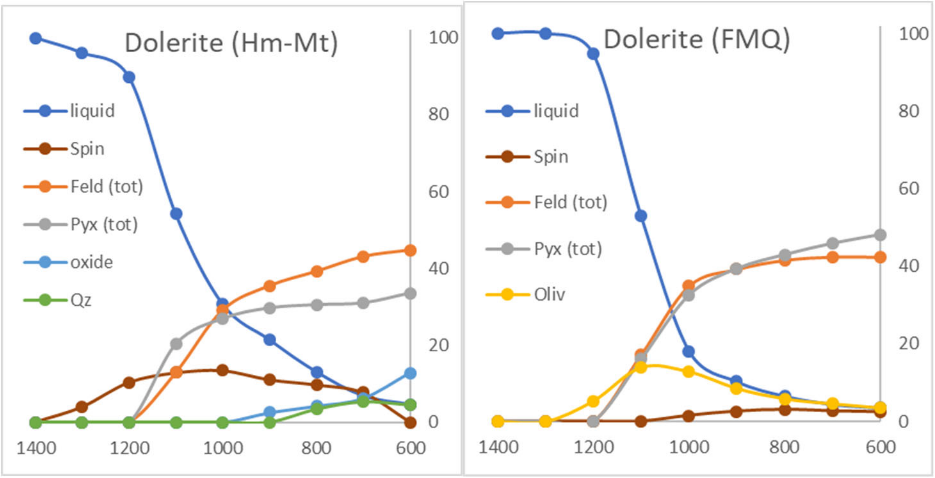

Fig. S-23. MELTS simulations for dolerite (dike)

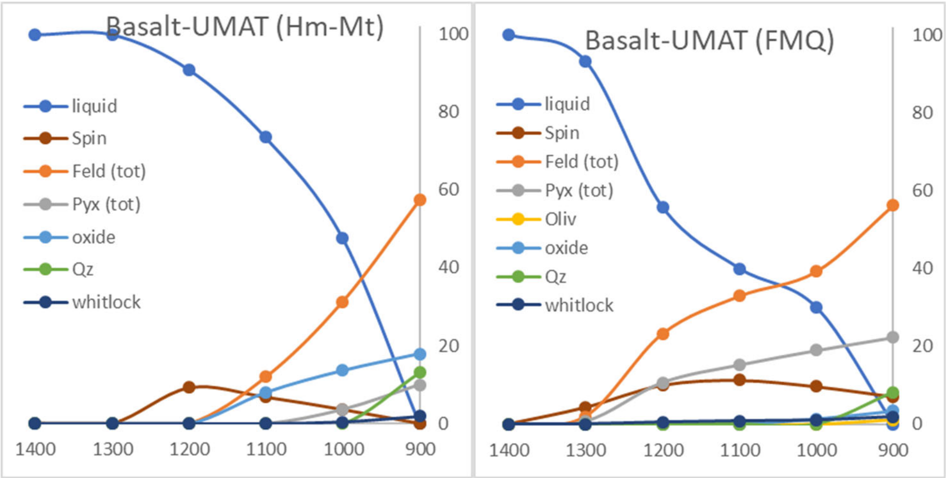

Fig. S-24. MELTS simulations for basalt (UMAT)

S6 Heat Treated Rocks – electron microscopy

See Methods section for interpretation of sample names. E.g., BA3-1200-P-L-Q indicates a chunk of BA3 amphibolite, heated at 1200°C for 15 min in a Pt crucible, and quenched in air.

S6.1 BA3 amphibolite

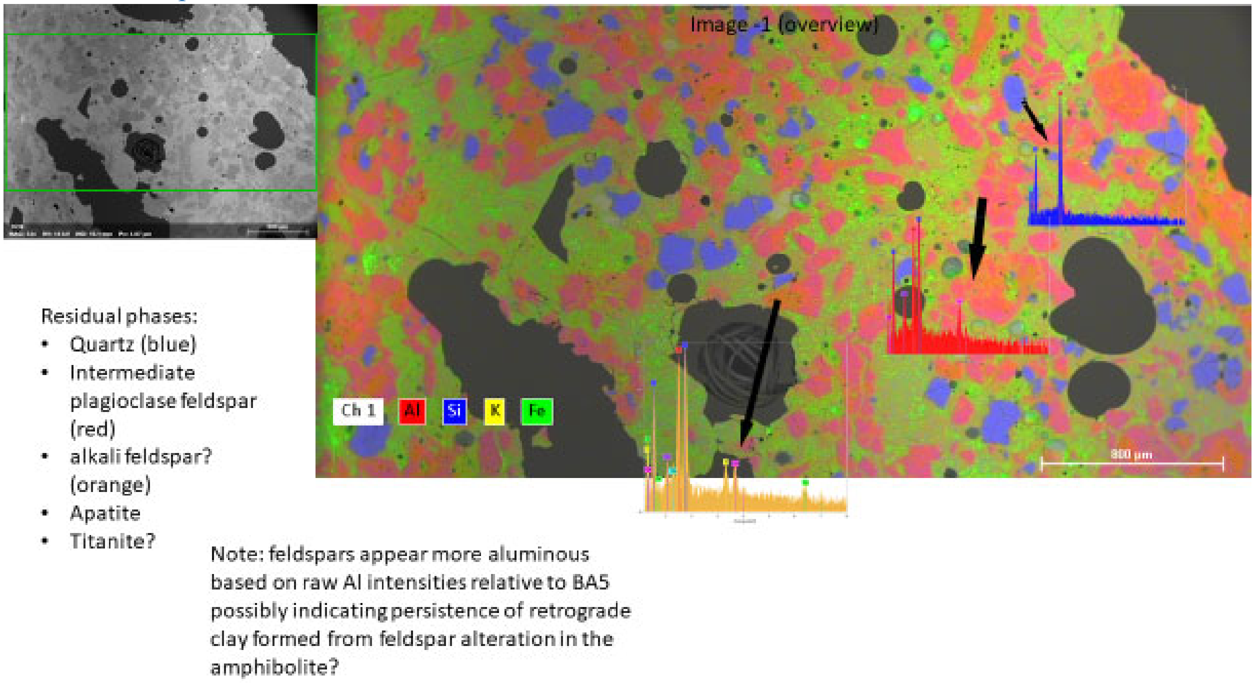

Fig. S-25. EDS composite image of BA3-1200-P-L-Q; obtained at Smithsonian MCI.

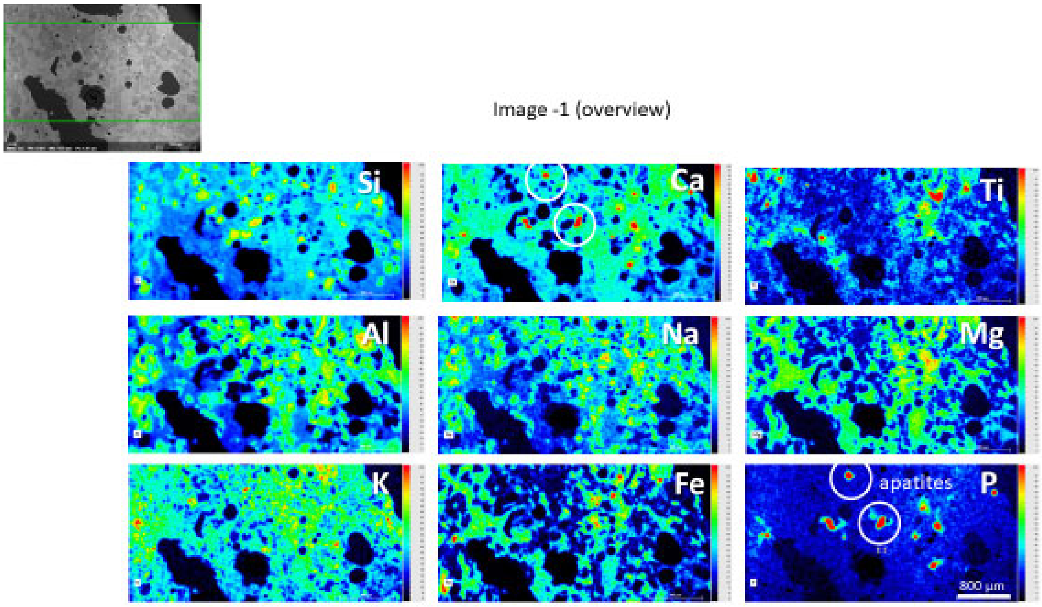

Fig. S-26. EDS maps of BA3-1200-P-L-Q; obtained at Smithsonian MCI. Red is high concentration and blue is low concentration.

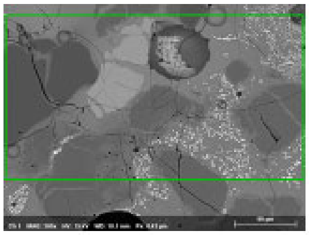

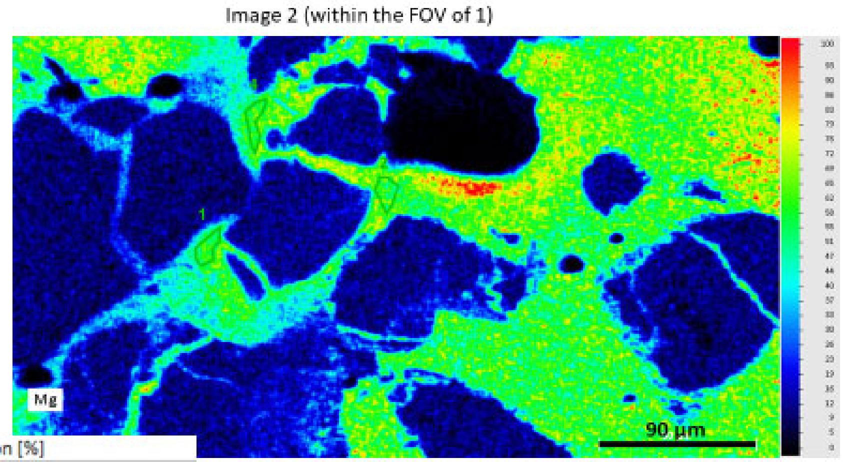

Estimate of glass composition

Normalized stoichiometric concentration [%]

Spectrum	C	O	Fe2O3	MgO	Al2O3	SiO2	CaO	K2O	Na2O
1.spx	0.00	0.00	10.23	5.64	11.96	52.04	11.59	1.79	2.21
2.spx	0.00	0.00	10.54	5.64	15.06	45.32	13.58	1.72	1.97
4.spx	0.00	0.00	10.69	5.61	13.71	45.57	14.62	1.64	1.99
Mean	**0.00**	**0.00**	**10.48**	**5.63**	**13.58**	**47.64**	**13.26**	**1.72**	**2.06**
Sigma	**0.00**	**0.00**	**0.23**	**0.02**	**1.55**	**3.81**	**1.54**	**0.08**	**0.14**

Low silica glass relative to BA5-1200 °C

Fig. S-27. EDS Mg map and glass composition of BA3-1200-P-L-Q; obtained at Smithsonian MCI. Red is high concentration and blue is low concentration.

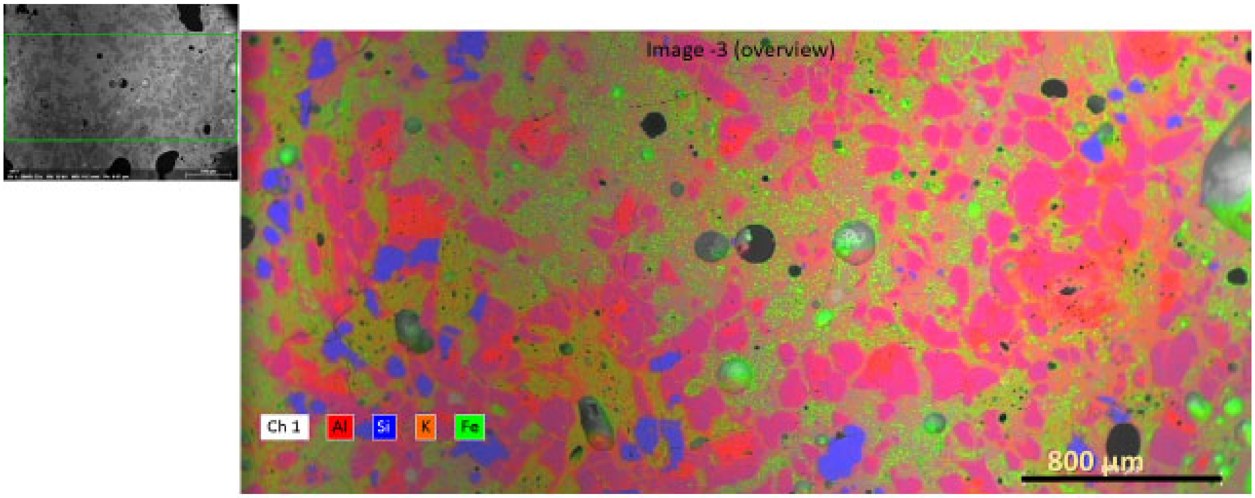

Fig. S-28. EDS composite image of BA3-1200-P-L-Q; obtained at Smithsonian MCI.

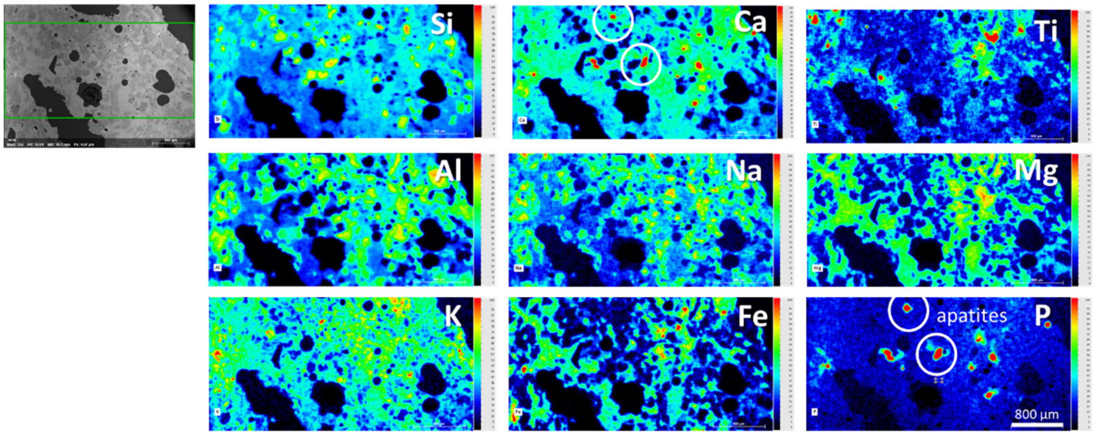

Fig. S-29. EDS maps of BA3-1200-P-L-Q; obtained at Smithsonian MCI. Red is high concentration and blue is low concentration.

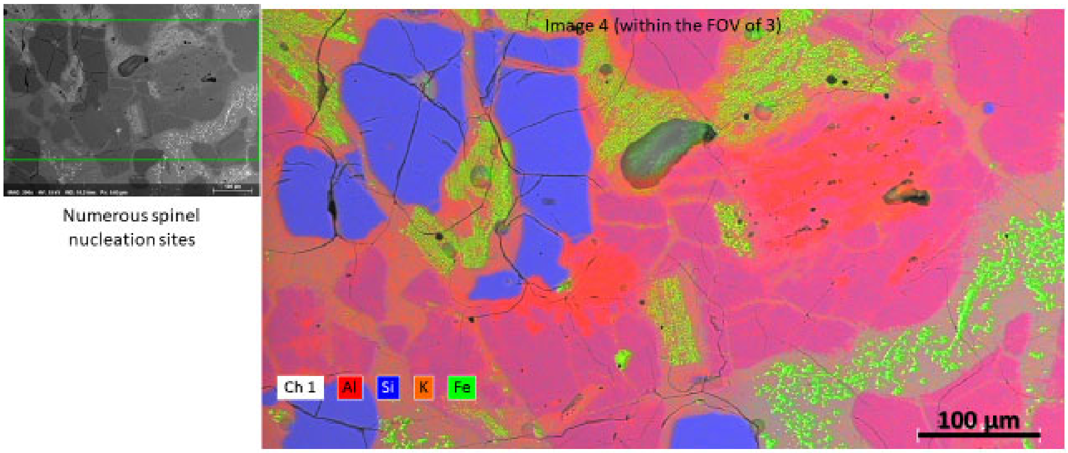

Fig. S-30. EDS composite image of BA3-1200-P-L-Q; obtained at Smithsonian MCI.

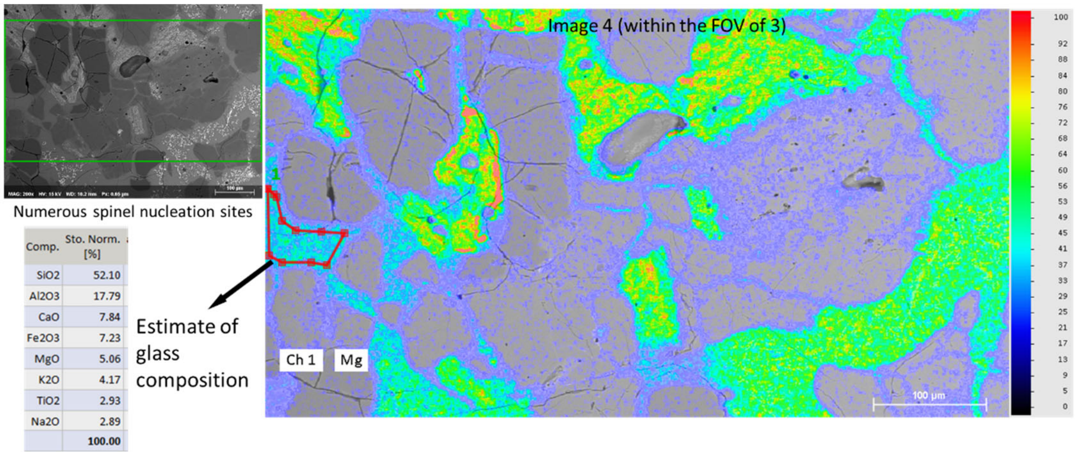

Comp.	Sto. Norm. [%]
SiO2	52.10
Al2O3	17.79
CaO	7.84
Fe2O3	7.23
MgO	5.06
K2O	4.17
TiO2	2.93
Na2O	2.89
	100.00

Fig. S-31. EDS Mg map of BA3-1200-P-L-Q and glass composition; obtained at Smithsonian MCI. Red is high concentration and blue is low concentration.

S6.2 BA5 amphibolite

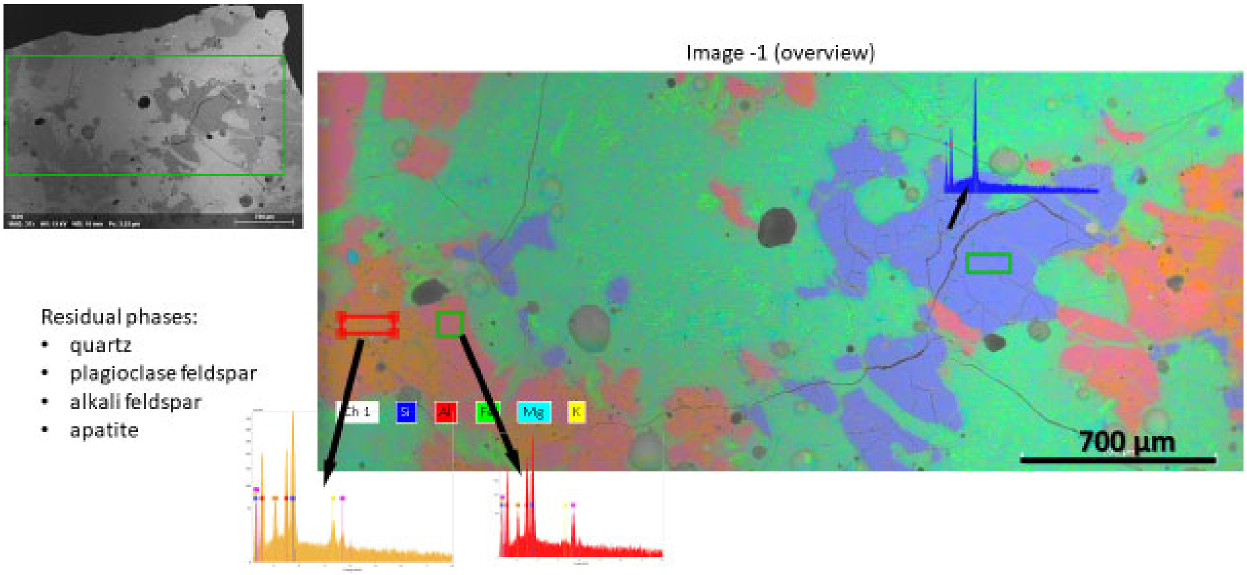

Fig. S-32. EDS composite image of BA5-1200-P-L-Q; obtained at Smithsonian MCI.

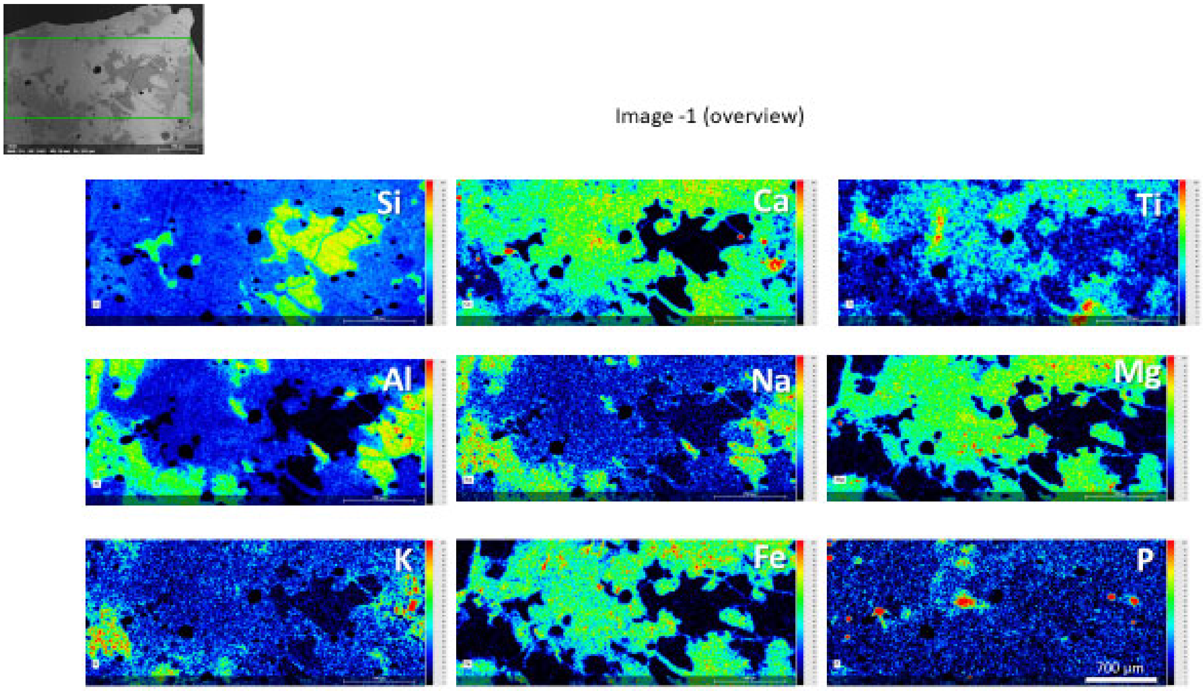

Fig. S-33. EDS maps of BA5-1200-P-L-Q; obtained at Smithsonian MCI. Red is high concentration and blue is low concentration.

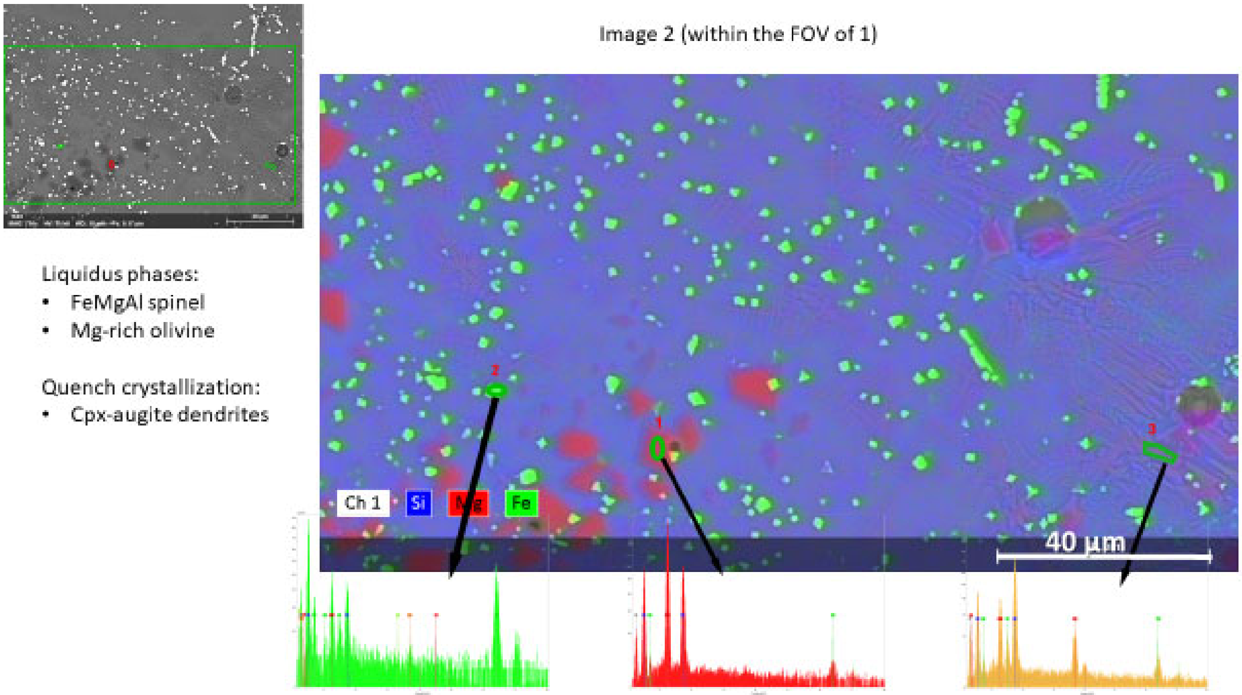

Fig. S-34. EDS composite image of BA5-1200-P-L-Q; obtained at Smithsonian MCI.

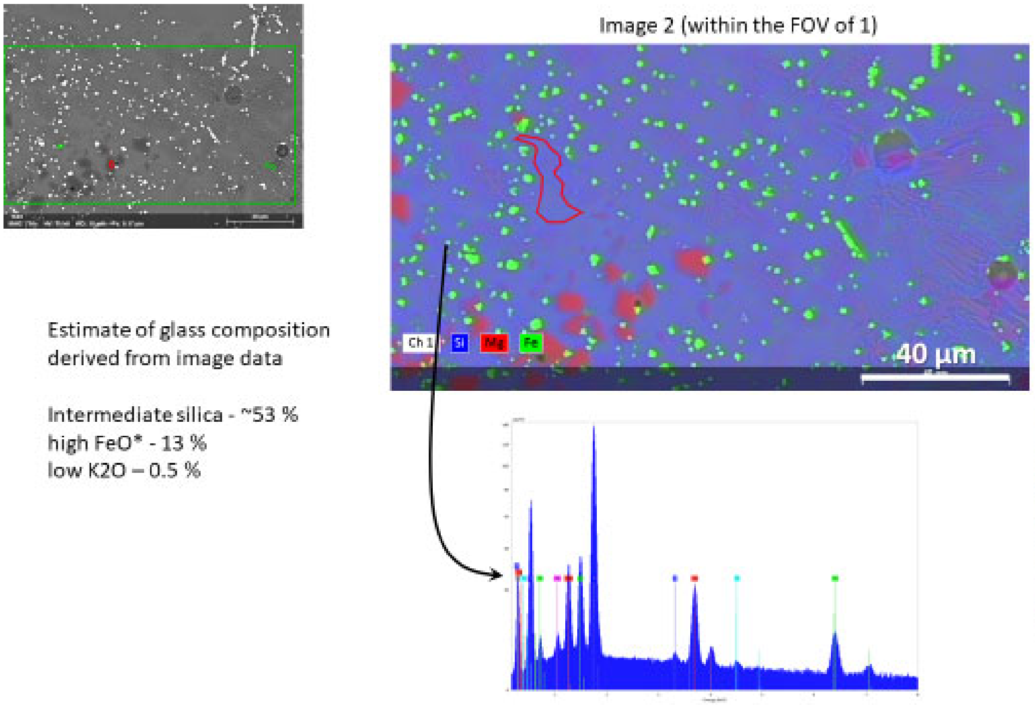

Comp.	Sto. Norm. [%]
	0.0
	0.0
SiO2	53.4
MgO	7.8
FeO	13.0
CaO	13.1
Al2O3	10.4
K2O	0.5
TiO2	0.7
Na2O	1.1
	100.0

Fig. S-35. EDS composite image and glass composition of BA5-1200-P-L-Q; obtained at Smithsonian MCI.

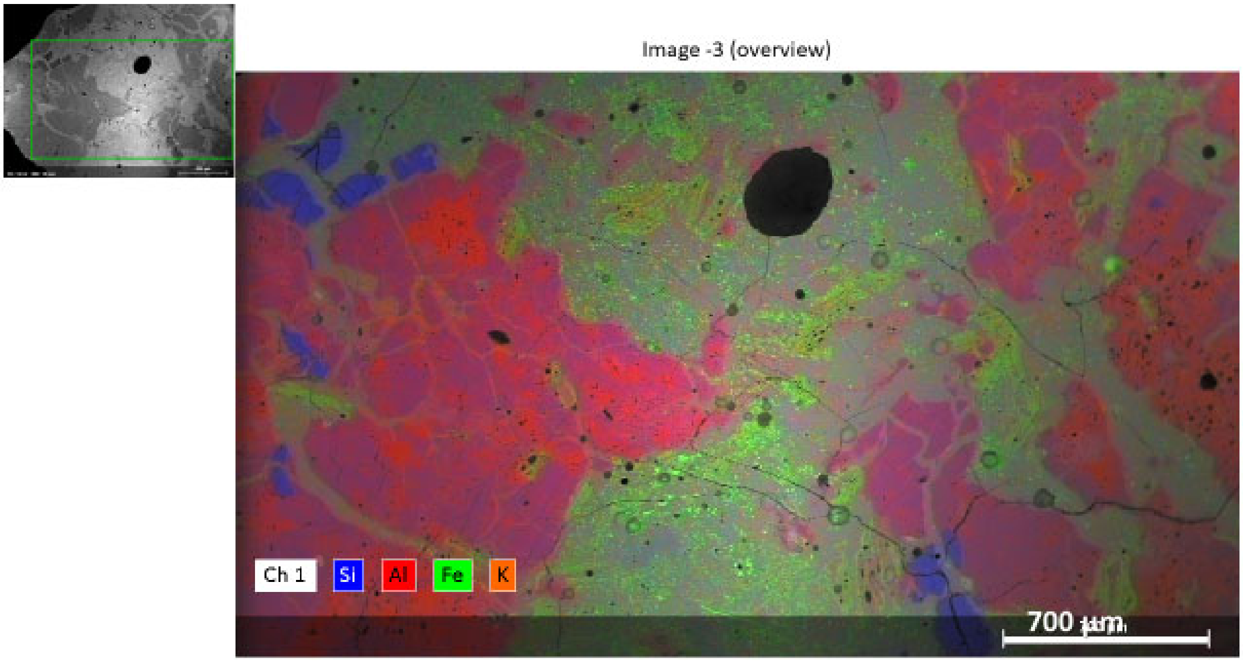

Fig. S-36. EDS composite image of BA5-1200-P-L-Q; obtained at Smithsonian MCI.

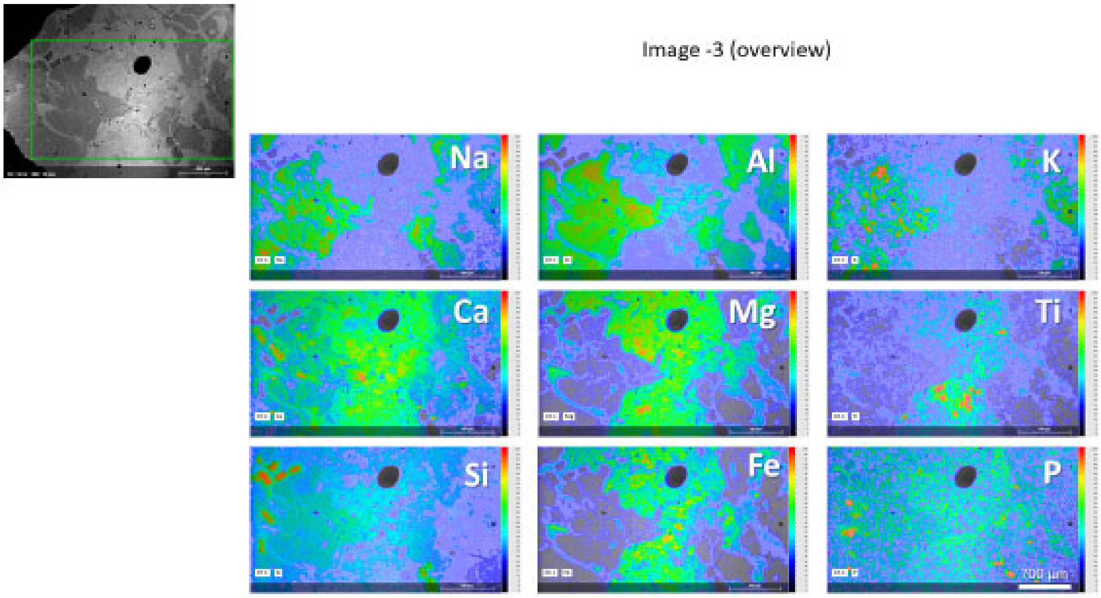

Fig. S-37. EDS maps of BA5-1200-P-L-Q; obtained at Smithsonian MCI. Red is high concentration and blue is low concentration.

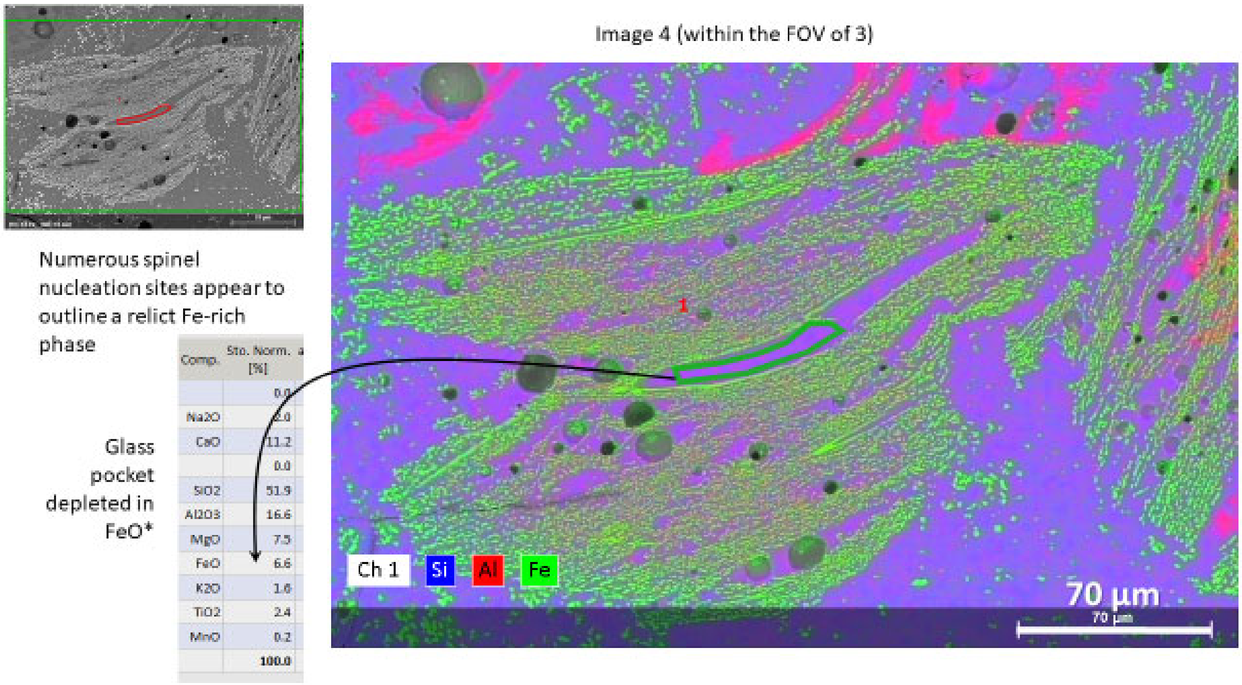

Comp.	Sto. Norm. [%]
	0.0
Na2O	2.0
CaO	11.2
	0.0
SiO2	51.9
Al2O3	16.6
MgO	7.5
FeO	6.6
K2O	1.6
TiO2	2.4
MnO	0.2
	100.0

Fig. S-38. EDS composite image and glass pocket composition of BA5-1200-P-L-Q; obtained at Smithsonian MCI.

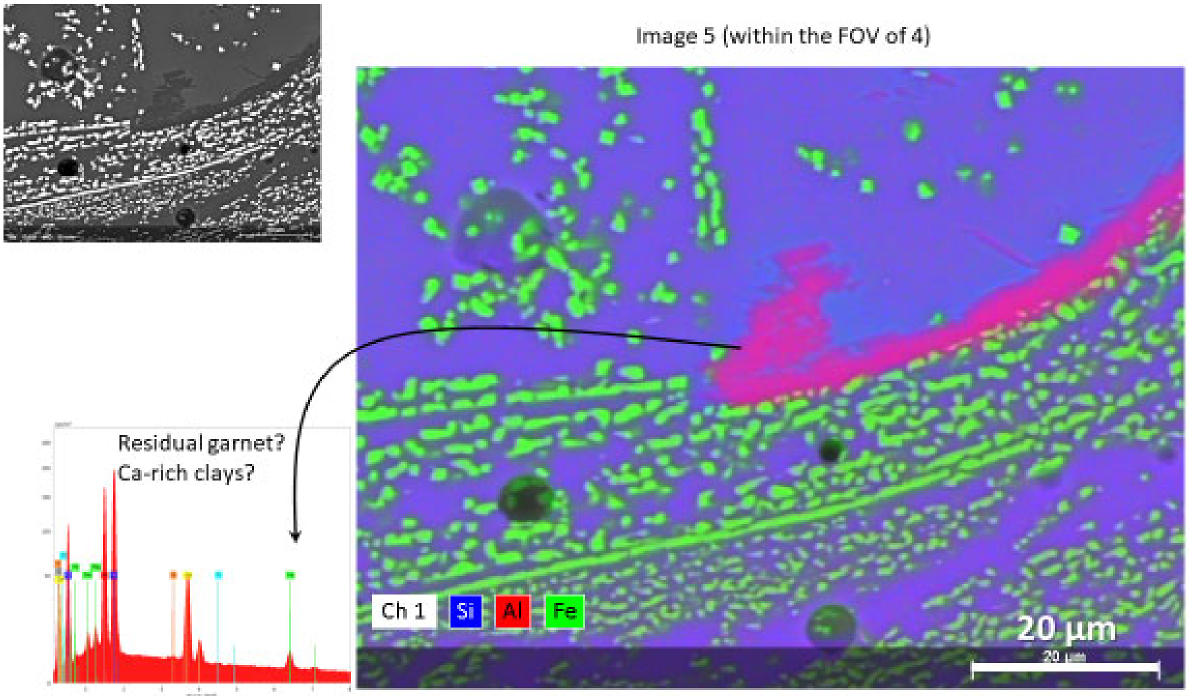

Fig. S-39. EDS composite image and spectrum in BA5-1200-P-L-Q; obtained at Smithsonian MCI.

S6.3 Granitoids

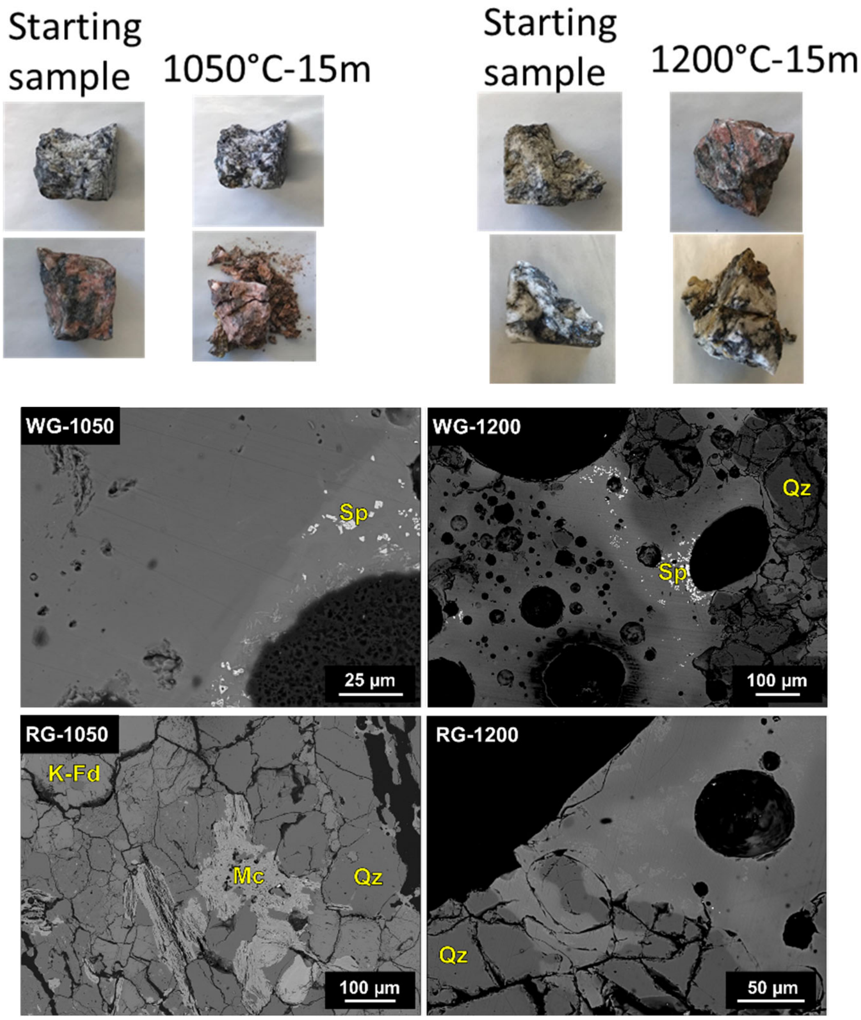

Fig. S-40. Optical images (top) and SEM backscattered images (bottom) of heat-treated source rocks: white gneiss (WG) and red gneiss (RG) at 1050°C and 1200°C, showing mostly glass with some crystals; spinel (Sp), quartz (Qz), K-feldspar (K-Fd), and mica (Mc). Glass compositions measured by EPMA are shown in Table 1 in the main text.

S7 References

S1. E. Ogenhall, Amphibolitic rocks near Broborg, Uppland, GAL PM 2016:03, The Archaeologists, National Historical Museums (SHM), Uppsala, Sweden, 2016.

S2. D. Lenaz and F. Princivalle, Crystal chemistry of clinopyroxenes from Oligo-Miocene volcanics of Montresta (Sardinia, Italy), *Mineral. Petrol.*, 2007, **90**, 157-166.

S3. S. A. T. Redfern, G. Artioli, R. Rinaldi, C. M. B. Henderson, K. S. Knight and B. J. Wood, Octahedral cation ordering in olivine at high temperature. II: an in situ neutron powder diffraction study on synthetic $MgFeSiO_4$ (Fa50), *Phys. Chem. Miner.*, 2000, **27**, 630-637.

S4. E. D. Zanotto and D. R. Cassar, The microscopic origin of the extreme glass-forming ability of Albite and B_2O_3, *Sci. Rep.*, 2017, **7**, 43022.
